# Benefits of asynchronous exclusion for the evolution of cooperation in stochastic evolutionary optional public goods games

**DOI:** 10.1038/s41598-019-44725-y

**Published:** 2019-06-03

**Authors:** Ji Quan, Junjun Zheng, Xianjia Wang, Xiukang Yang

**Affiliations:** 1 School of Management, Wuhan University of Technology, Wuhan, 430070 China; 2 School of Economics and Management, Wuhan University, Wuhan, 430072 China

**Keywords:** Evolutionary theory, Computational science

## Abstract

Mechanisms and conditions for the spontaneous emergence of cooperation in multi-player social dilemma games remain an open question. This paper focuses on stochastic evolutionary optional public goods games with different exclusion strategies. We introduce four strategy types in the population, namely, cooperation, defection, loner and exclusion. Synchronous and asynchronous exclusion forms have been compared in finite-sized, well-mixed and structured populations. In addition, we verify that the asynchronous exclusion mechanism is indeed better than the synchronous exclusion mechanism in all cases. The benefits of the asynchronous exclusion are measured by comparing the probability that the system chooses the cooperative states in the two situations. In the well-mixed population cases, only when the investment amplification factor is small and the probability of exclusion success is high will the asynchronous exclusion mechanism have a relatively large advantage in promoting cooperation. However, in the structured population cases, the range of the investment amplification factor, in which the asynchronous exclusion mechanism has relatively large advantages in promoting cooperation, is somewhat different and is mainly in the middle of the interval under our parameters. Our study further corroborated that when non-participation and exclusion strategies exist, a structured population does not necessarily promote cooperation compared with a well-mixed population for some parameter combinations. Thus, we acquire a good understanding of the emergence of cooperation under different exclusion mechanisms.

## Introduction

Social dilemma means that individual rationality can lead to collective irrationality^1–3^. The issue of cooperation between multiple subjects is a typical type of social dilemma^4^. On the one hand, the dilemma of cooperation is prevalent in the social and economic activities of multiple people and multiple organizations^5–7^. On the other hand, the phenomenon of cooperation is everywhere in actual social relationships^8–10^. Cooperation is the foundation of human social progress and civilization, and most of the cooperation in reality is spontaneous in the absence of centralization. Thus, the following questions are raised: (1) Under what conditions can cooperation be spontaneously emerging from self-interested individuals without centralized power? (2) What is the mechanism of cooperation when everyone has selfish motives? In fact, these issues have plagued theoretical scientists for many years^11,12^.

Currently, many scholars from different disciplines have studied the evolution of human cooperative behaviours^6,9,10,13–20^. A few mature research frameworks and methodologies are now in place^21–25^, and certain mechanisms to interpret cooperation have also been proposed^24,26–32^. From the behavioral science perspective, the issue of cooperation is essentially an incentive problem^33,34^. Hence, if we want to achieve collective rationality by individual choice and obtain the benefits of cooperation, then we must motivate and induce individual behaviours. The examples are punishing uncooperative behaviours^29,35–39^ or rewarding cooperative behaviours^33,40–43^. However, in reality, punishments and rewards are costly, the process of which is essentially a second-order social dilemma^44–46^. Thus, who will impose punishments or rewards? How are punishments or rewards implemented? These questions have become the core issues in this incentive problem.

If punishment or reward is used as a type of strategy for the individuals, then, the conditions under which cooperative strategies can be emerging through systematic self-organization can be explored. Presently, various forms of punishments or rewards have been proposed under the direct reciprocity mechanism^36,37,43,47–61^. An example is introducing punishing strategies to punish cooperators and defectors^52,59^; introducing the moralists who cooperate whilst punishing non-cooperative behaviours and immoralists who defect whilst punishing other non-cooperative behaviours^60^; self-organised punishment that allows players to adapt their punishment depending on the frequency of cooperation^49^; tolerance-based punishments in which individuals punish their co-players based on social tolerance^61^; conditional punishments with fine depending on the number of punishers^47^; implicated punishments that punish all individuals in the group once a evildoer is caught^56^; probabilistic sharing the cost of punishing defectors^48^; and heterogeneous punishments that group punishers based on their willingness to bear the punishing^57^. Perc *et al*.^10^ presented a thoroughly review on punishment mechanisms in evolutionary games. Notably, in the real world, a few shortcomings exist for punishment in promoting cooperation^62^. Apart from the efficiency loss that may be caused by implementing this behaviour, the promotion effect may also be affected by other factors owing to the increase in the process. For instance, Nikiforakis *et al*.^63^ corroborated that the form of punishment and its feedback in the public goods game will have an impact on group cooperation behaviour through behavioural experiments, and only appropriate punishment feedback can promote cooperation in the population.

In the traditional form of punishment, the punishing strategy must generally pay a cost, and individuals who are punished must simultaneously pay a larger penalty cost. The exclusion strategy, which has been proposed recently, can be regarded as a new form of punishing strategy^64^. Unlike the traditional assumption that defectors who are punished must pay a fixed penalty cost, it assumes that defectors will be expelled from the group by excluders with a certain probability. Moreover, the deported individuals cannot share the cooperative benefits of the group. This mechanism has received widespread attention since its introduction^65–67^. For instance, Li *et al*.^66^ extended the evolutionary public goods game model with exclusion strategies to a finite size population for the first time. Further, Liu *et al*.^67^ simultaneously introduced prosocial punishment and exclusion type strategies and studied competitions between them, and they affirmed that exclusion can outperform punishment when they coexist. What’s more, Li *et al*.^68^ proposed the concept of sequential exclusion, also called asynchronous exclusion, and they contended that asynchronous exclusion is a more effective mechanism than synchronous exclusion when three strategy types exist, namely, cooperation, defection and exclusion. Whether this conclusion remains valid when other strategy types or structured populations are introduced requires investigation. In addition, how to measure the advantage of the asynchronous exclusion mechanism and how the advantage is affected by the system parameters entail further studies. Accordingly, this study aims to answer such arguments.

This study introduces four strategy types used in evolutionary public goods games in two population types, namely, finite-sized, well-mixed and structured populations. In addition, synchronous and asynchronous exclusion forms have been considered. In the well-mixed interactive population, similar to literature^37,69^, the population state is described by a Markov process. Different from them, the competitive evolution between different strategies is introduced. Based on the same evolutionary dynamic model, the stochastic stable equilibria of the system in the two exclusion cases and the effects of parameters on the probabilities of the system choosing different equilibria are analyzed. Thus, the benefits of the asynchronous exclusion mechanism can be measured by comparing the probability that the system chooses the cooperative states in the two situations. In the structured population, we also compare the evolutionary stable state of the system under the two exclusion mechanisms. Hence, the effects of parameters on the frequency of strategies in the stable state in the two exclusion situations can be obtained. We provide the exact range of parameters, which makes the asynchronous exclusion mechanism relatively efficient. Ultimately, these results elucidate the emergence of cooperation under different exclusion mechanisms.

## Results

### Synchronous and asynchronous exclusion in optional public goods games

We introduce four types of individuals in the public goods game (PGG), namely, cooperation (denote as *C*), defection (denote as *D*), non-participation (denote as *L*) and exclusion (denote as *E*). Cooperation type individuals invest in the public goods and share the benefits of their investment income, whereas defection type individuals do not invest in the public goods but can take a free ride of the cooperators’ investment income. Without the loss of generality, we let the investment cost equals one. Non-participation type individuals, who are typically called loners, do not take part in the game but can obtain a fixed income *σ*, whereas exclusion type individuals not only participate in the investment in the public goods game, but also exclude defectors in the group. The exclusion behaviour is costly, which will bring additional cost to the excluders. However, it can prevent free-riders from sharing their investment income. The defectors who are excluded from the group cannot share the benefit of the public goods. In addition, we assume that the exclusion behaviour cannot successfully exclude defectors with certainty but only with probability *β*. Let *c*_*E*_ denote the unit exclusion cost for an excluder. Evidently, the higher the probability *β*, the greater the cost *c*_*E*_; thus, we assume that *c*_*E*_ is a function of *β* with ${c}_{E}^{^{\prime} }(\beta ) > 0$ and ${c}_{E}^{^{\prime\prime} }(\beta ) > 0$. Furthermore, this study considers two types of exclusion mechanisms. The first is synchronous exclusion, under which each individual of the exclusion type independently and simultaneously expels all defectors. The other is asynchronous exclusion, and the exclusion process is sequential, which means that once a defector is expelled by an excluder, the latter excluders no longer have to spend the exclusion cost for this defector. Other parameters in our model are as follows. *M* is the population size (number of individuals). *r* is the amplification coefficient of the *N*-person public goods game (1 < *r* < *N*). *κ* is a parameter to describe individuals’ reaction speed to the environment in their decision.

### Numerical experiments in a finite size and well-mixed population

In this situation, each individual interacts with others with equal probability. Each time, *N* individuals are randomly sampled from the population to participate in the PGG. We focus on the stochastic stable states of the evolutionary system and the corresponding probability of the system to choose each stable state. We aim to compare the differences in the probability of the system selecting each evolutionary steady state under the two different exclusion mechanisms. By a large number of numerical experiments under arbitrary parameters in both situations, only the states of (0, *M*, 0, 0), (0, 0, *M*, 0), (0, 1, *M* − 1, 0), (0, 0, *M* − 1, 1) and (*i*, 0, 0, *M* − *i*) (0 ≤ *i* ≤ *M*) may be stochastically stable, where state (*i*, *j*, *k*, *l*) denotes the number of cooperators, defectors, loners and excluders, respectively, in the population. For example, (0, *M*, 0, 0) indicates that all individuals choose the defection strategy, and we denote it as the ‘All D’ state. Similarly, (*i*, 0, 0, *M* − *i*) corresponds to the ‘C + E’ states. According to the model assumption, when *N* − 1 loners exist in the sampled group, the other individual can only obtain a fixed payoff, regardless of its type, which is equivalent to all individuals being loners. Therefore, states (0, 0, *M*, 0),(0, 1, *M* − 1, 0) and (0, 0, *M* − 1, 1) can be collectively referred to as the ‘All L’ state. In the following, we fix parameters *M* = 20, *N* = 5, *κ* = 1 and let *c*_*E*_ = 0.2 * 10^*β*^ to study the effects of parameters *β*, *r*, *σ* on the probability of the system to select each stable equilibrium. We provide results for larger size populations (*M* = 50 and 100) in Supplementary Information. The probability that the system selects some stable states will change significantly when *M* increases to 100, but for the main conclusions we present, there is no essential difference between *M* = 100 and *M* = 20.

**Figure 1:**
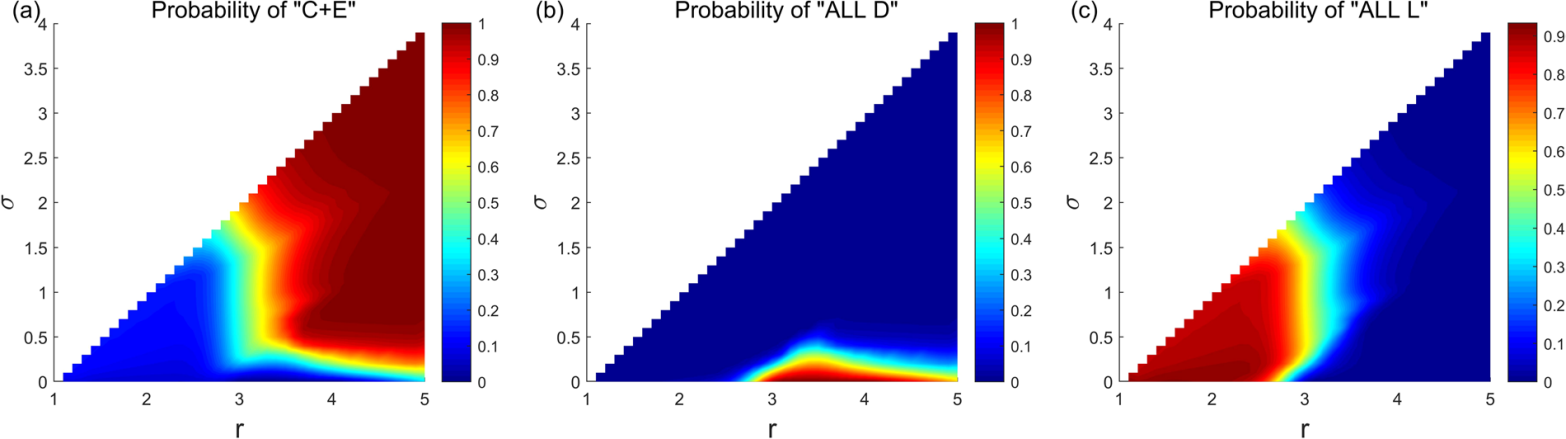
Limit probability of the system selecting each stochastic stable state under any parameter combinations of (*r*, *σ*) and fixed *β* = 0.1 in the asynchronous exclusion. In this situation, a large parameter region D_1_ exists, leading the system to select the cooperative state with a low probability. Moreover, a corresponding region D_2_ exists, leading the system to select the ‘All L’ state with a high probability. Notably, a small parameter area D_3_ also exists corresponding to large values of *r* and small values of *σ*, which leads the system to select the ‘All D’ state with a high probability.

**Figure 2:**
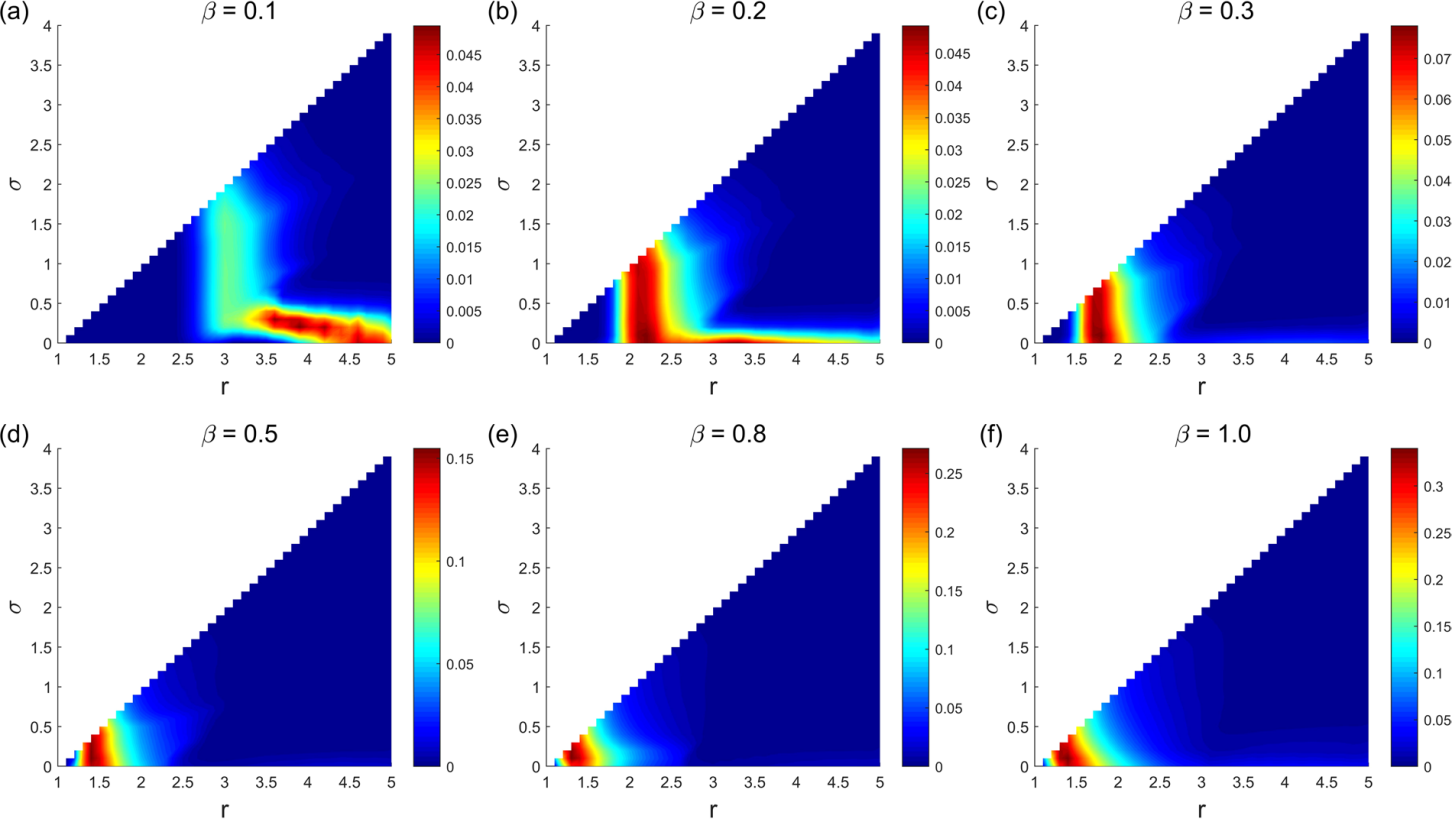
Relationship between the probability difference of the system selecting the cooperative states and parameters (*r*, *σ*) under the two exclusion mechanisms. (**a**) *β* = 0.1; (**b**) *β* = 0.2; (**c**) *β* = 0.3; (**d**) *β* = 0.5; (**e**) *β* = 0.8; (**f**) *β* = 1. When *β* is small, the probability difference of the system selecting the cooperative states under the two exclusion mechanisms is small. Moreover, the points with relatively large differences in probability are located in a small region that changes with the increase in *β*. Given that the maximal probability difference does not exceed 0.1 when *β* ≤ 0.3, the advantage of the asynchronous exclusion mechanism is not evident. As *β* increases, the maximal probability difference also increases, and the benefits of the asynchronous exclusion mechanism slowly emerge.

**Figure 3:**
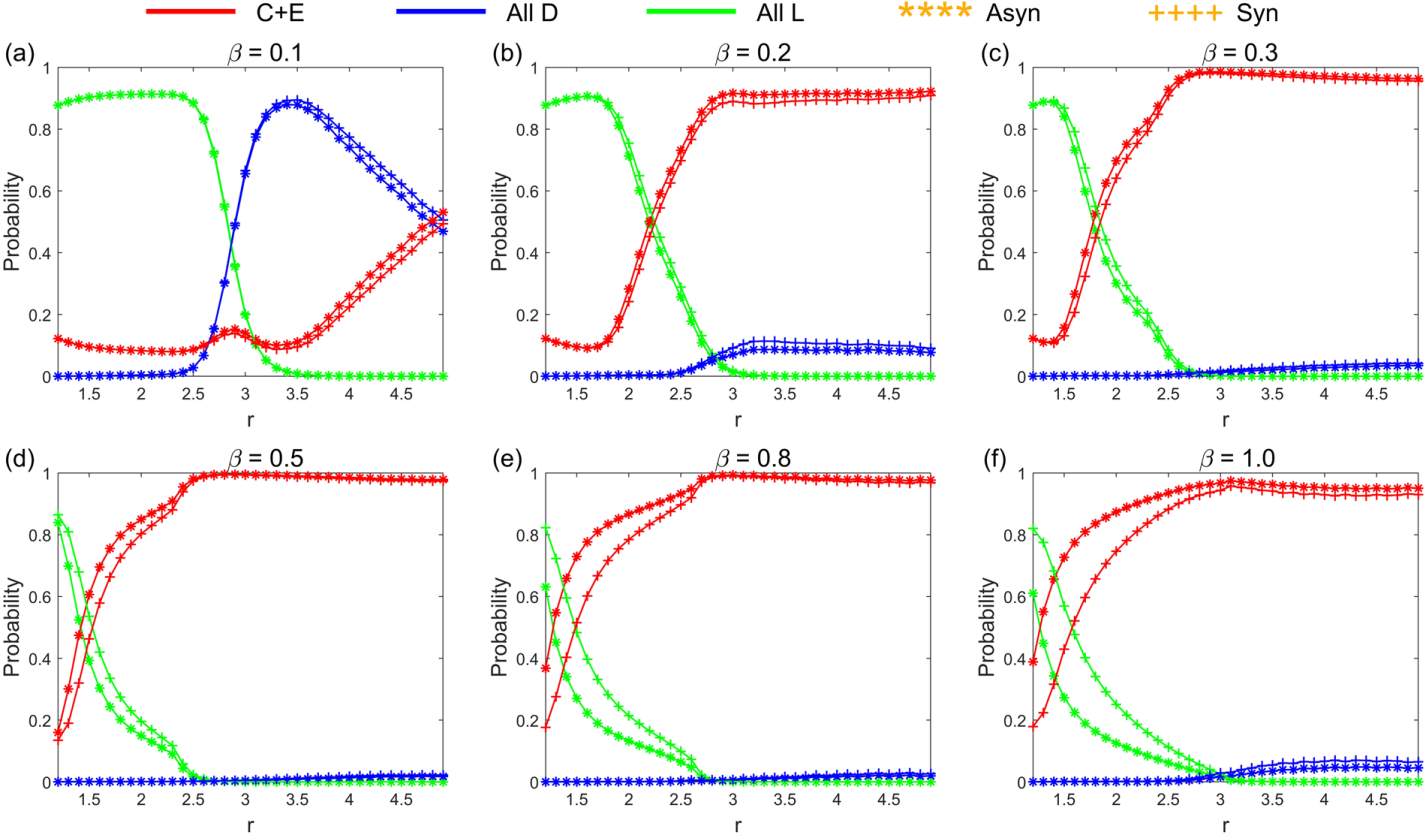
Relationship between the limit probability of the system selecting each type of stable states and parameter *r* for fixed *σ* = 0.1 under the two exclusion mechanisms. (**a**) *β* = 0.1; (**b**) *β* = 0.2; (**c**) *β* = 0.3; (**d**) *β* = 0.5; (**e**) *β* = 0.8; (**f**) *β* = 1. The values of *β* in the first three subgraphs are relatively small, and the advantages of the asynchronous exclusion mechanism are not evident. The values of *β* in the latter three subgraphs are relatively large, and when *r* is small, the benefits of the asynchronous exclusion mechanism emerge. It verifies that only when *r* is small and *β* is large will the asynchronous exclusion mechanism have a relatively large advantage in promoting cooperation.

**Figure 4:**
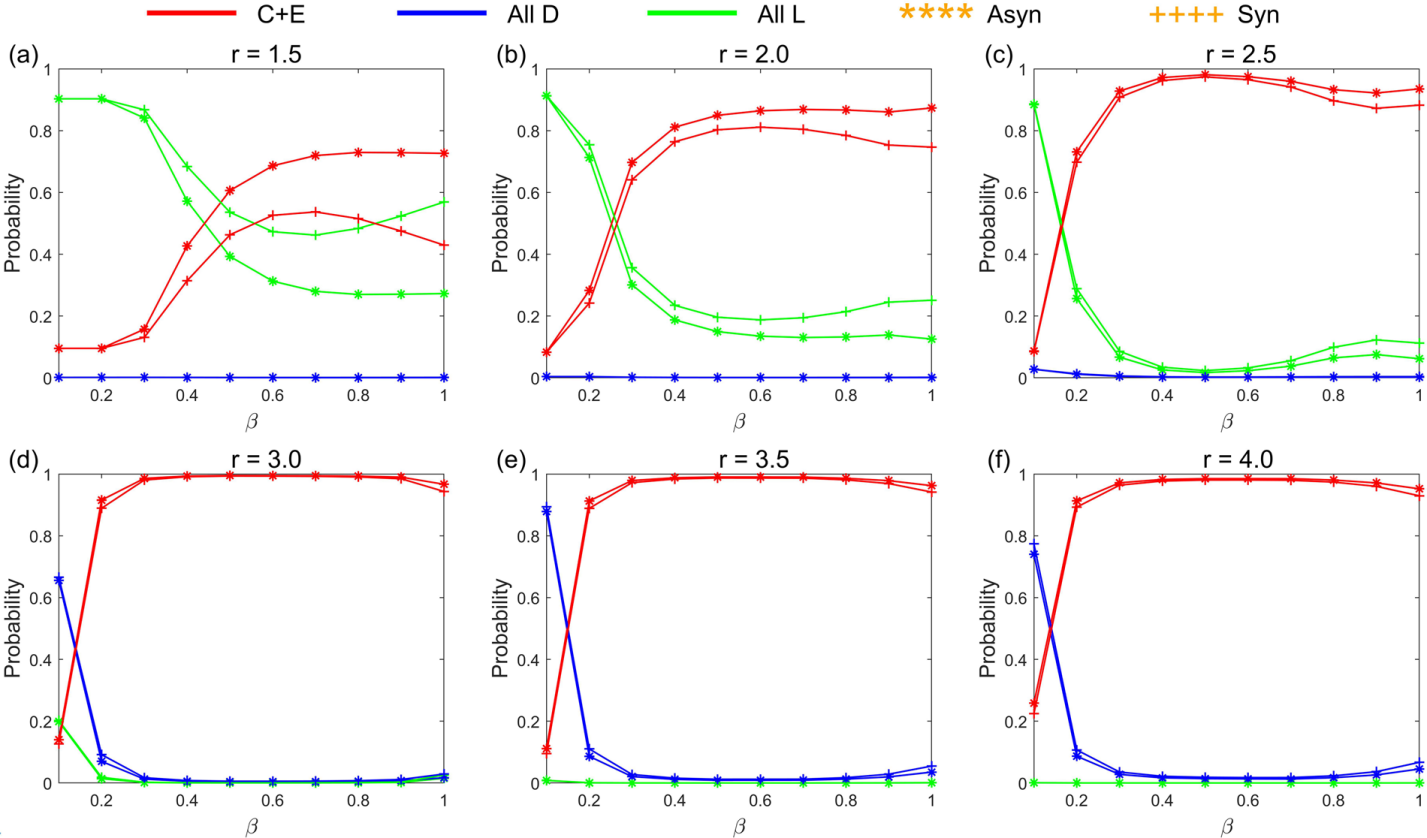
Relationship between the limit probability of the system selecting each type of stable state and parameter *β* for fixed *σ* = 0.1 under the two exclusion mechanisms. (**a**) *r* = 1.5; (**b**) *r* = 2.0; (**c**) *r* = 2.5; (**d**) *r* = 3.0; (**e**) *r* = 3.5; (**f**) *r* = 4. The values of *r* in the first two subgraphs are relatively small, and when *β* is large, the advantages of the asynchronous exclusion mechanism emerge. The values of *r* in the latter four subgraphs are relatively large, and the benefits of the asynchronous exclusion mechanism are not evident.

Figure 1 shows the limit probability of the system selecting each stochastic stable state under any parameter combinations of (*r*, *σ*) and fixed *β* = 0.1 in the asynchronous exclusion. It depicts that when *β* is small, a large range of parameters (*r*, *σ*) (denote this region as D_1_) exists, in which the system selects the cooperative state with a low probability. Moreover, a corresponding region (denote as D_2_) exists, leading the system to select the ‘All L’ state with a high probability. Notably, a small parameter area (denote as D_3_) also exists corresponding to the large values of *r* and small values of *σ*, which leads the system to select the ‘All D’ state with a high probability. Notably, we notice that when *β* slowly increases, all three regions shrink rapidly. Moreover, the probability of the system selecting the ‘All D’ state also drop rapidly when parameters (*r*, *σ*) are in the D_3_ region. More details can be found in Supplementary Information. The results are somewhat similar in the synchronous exclusion situation. However, the probability of the system reaching the cooperative state is lower than that of the asynchronous exclusion mechanism under the same combination of parameters. This finding further illustrates that the asynchronous exclusion mechanism works better for the evolution of cooperation. We use the probability difference to represent the benefit of asynchronous exclusion in promoting cooperation. Figure 2 shows the relationship between the probability difference and parameters (*r*, *σ*) when *β* is fixed at six different values, and it elucidates that when *β* is small, the probability difference of the system selecting the cooperative states under the two exclusion mechanisms is small. Moreover, the points with relatively large differences in probability are located in a small region D_4_ (D_4_ changes with the increase in *β*). Given that the maximal probability difference does not exceed 0.1 when *β* ≤ 0.3, the advantage of the asynchronous exclusion mechanism is not evident. As *β* increases, the maximal probability difference also increases, and the benefits of the asynchronous exclusion mechanism slowly emerge. Further, the points with relatively large differences in probability are located in an area with small values of *r* and small values of *σ*. To clearly compare the differences between the two exclusion situations, we fix the parameter *σ* = 0.1 to show the corresponding results. Figures 3 and 4 show the relationship between the limit probability of the system selecting each type of stable states and parameters *r* and *β*, respectively, for fixed *σ* = 0.1 under the two exclusion mechanisms. Only when *r* is small and *β* is large will the asynchronous exclusion mechanism have a relatively large advantage in promoting cooperation which is consistent with our theoretical analysis. In fact, when the number of excluders in the population is *l*, the expected unit exclusion costs in the asynchronous situation *c*_R_ and in the synchronous situation *c*_E_ satisfy the following equation: ${c}_{{\rm{R}}}=\frac{1-{(1-\beta )}^{l+1}}{(l+1)\beta }{c}_{E} < {c}_{E}$. The difference between *c*_R_ and *c*_E_ increases as *β* increases. Moreover, when *β* → 0^+^, no difference exists between the two costs.

### Simulation results in a structured population

**Figure 5:**
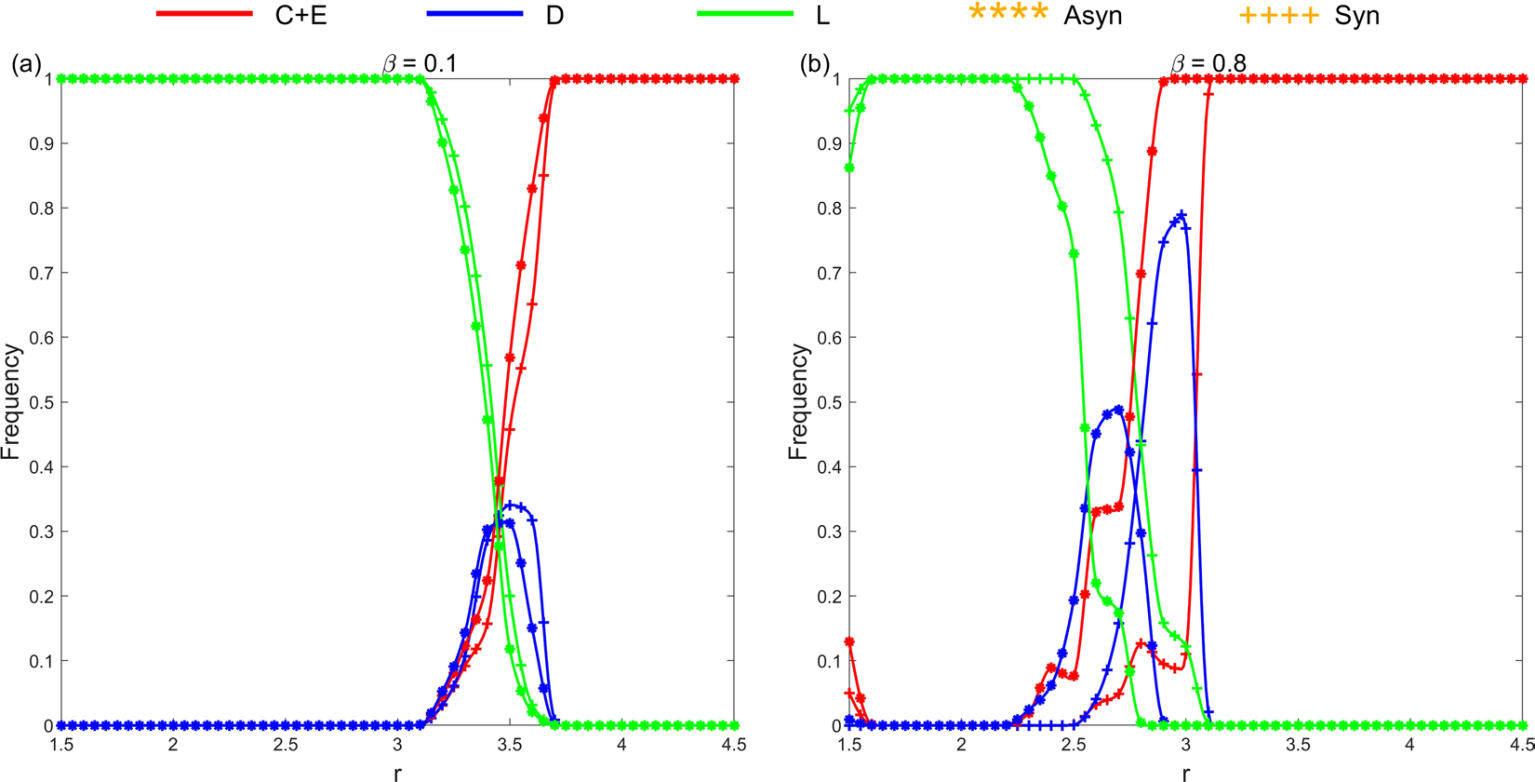
Relationship of the frequencies of all the strategy types in the squared lattice and parameter *r* for fixed *σ* = 0.1 after the system reaches evolutionary stability under the two exclusion mechanisms. (**a**) *β* = 0.1; (**b**) *β* = 0.8. When *β* = 0.1, the advantage of the asynchronous exclusion is not evident, and a common *r* interval (3.1, 3.7) exists in the two exclusion situations, so that the *D* strategy can emerge and the frequency of *D* peaks when *r* = 3.5 after the system becomes stable. When *β* = 0.8, the asynchronous exclusion mechanism has a significant advantage mainly in the middle interval of *r*. Two different *r* intervals (2.2, 2.9) and (2.5, 3.1) exist, which correspond to the asynchronous and synchronous exclusions, respectively, so that the *D* strategy can emerge.

In this situation, each individual is located on a node of the square lattice, and plays the PGG with its four direct neighbors. Thus, each individual participates in five rounds of games to accumulate payoff. Exclusion individual *E* will exclude their adjacent defectors with certain probability *β* for each, whilst paying *β*-related unit cost *c*_*E*_. Defectors who are expelled from the group cannot obtain investment income in the corresponding single-round game. Moreover, we consider synchronous and asynchronous exclusion situations. Figure 5 shows the frequencies of all strategy types after the system reaches evolutionary stability in the two exclusion situations under two values of *β* = 0.1 and 0.8, respectively, when *r* changes from 1.5 to 4.5. For each simulation, the system iterates 10,000 to 50,000 rounds (10,000 times for a round), ensuring that the system has reached an evolutionary stable state. We take the average of the last 1000 rounds and obtain all the simulation results by the average results of 20 independent simulation experiments.

**Figure 6:**
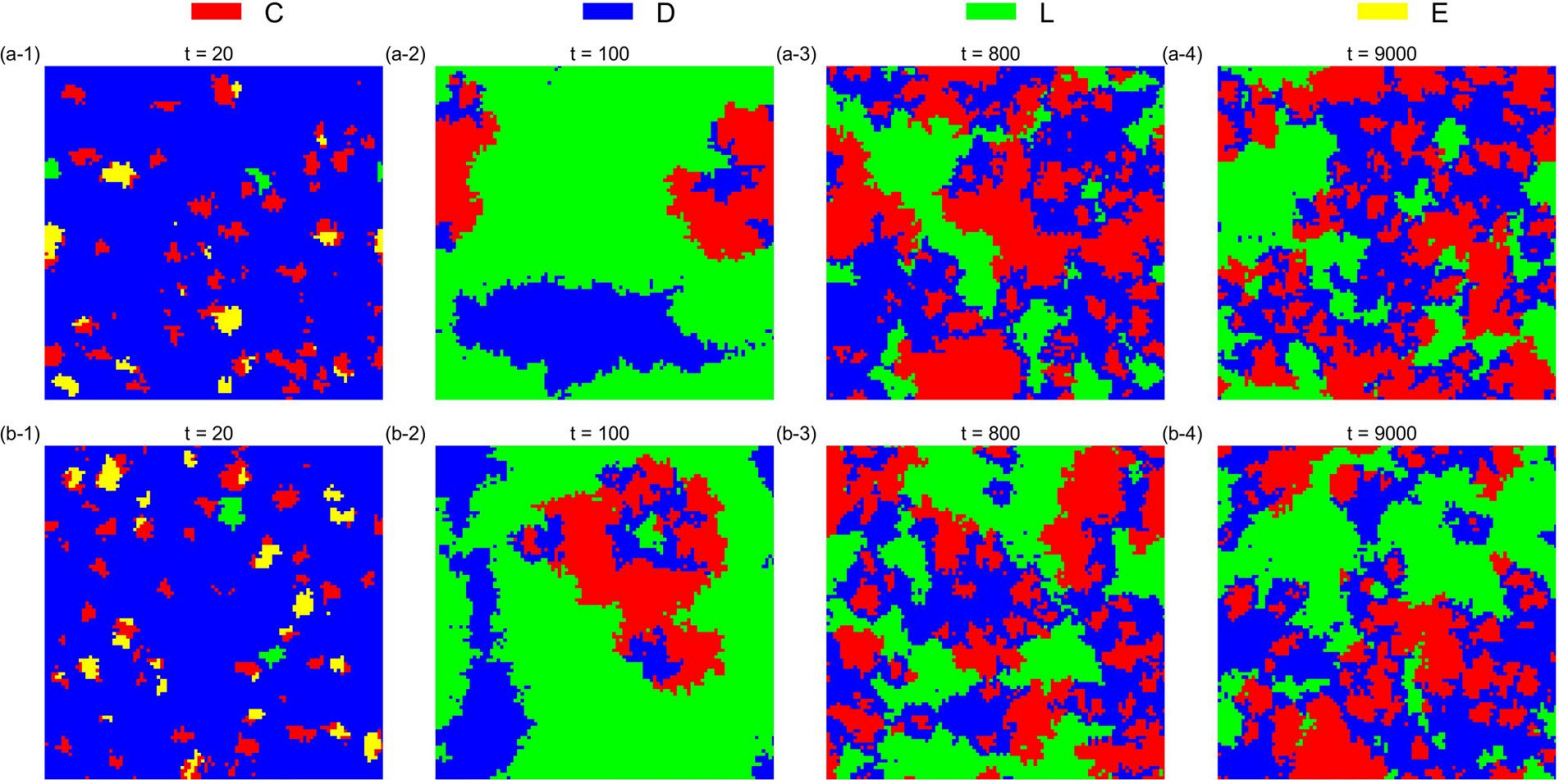
Spatiotemporal distribution of the four strategies in the PGG at *t* = 20,40,60,300 Monte Carlo steps (MCS) and *t* = 20,200,2000,5000 MCS, respectively, in the two exclusion situations for one simulation, when *β* = 0.1, *r* = 3.5 and *σ* = 0.1. (**a**) Synchronous exclusion; (**b**) asynchronous exclusion. In both exclusion situations, the system reaches the stable state of C + D + L, that is, the three strategies coexist.

**Figure 7:**
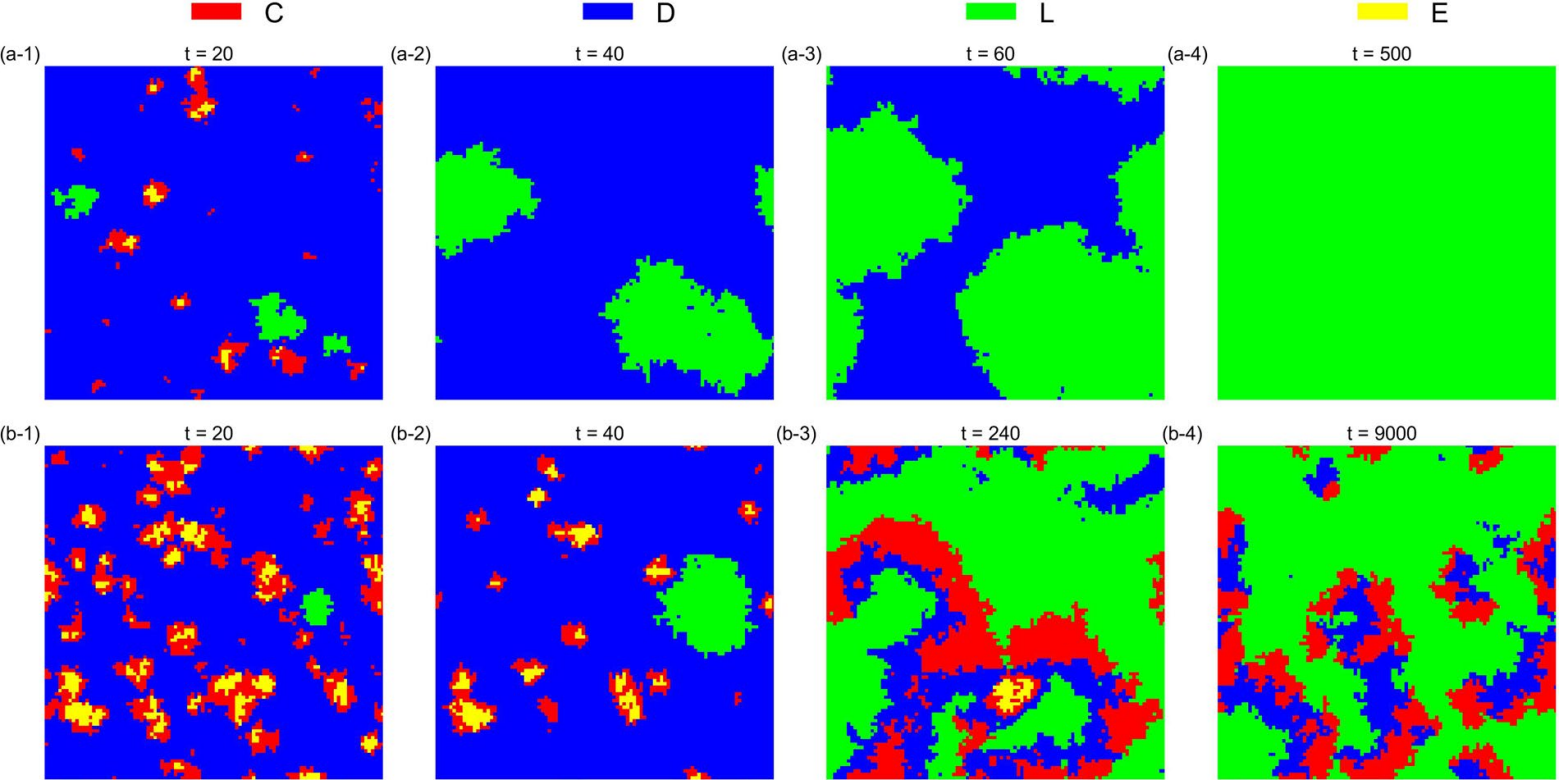
Spatiotemporal distribution of the four strategies in the PGG at *t* = 20, 40, 60, 500 MCS and *t* = 20, 40, 240, 9000 MCS, respectively, in the two exclusion situations for one simulation, when *β* = 0.8, *r* = 2.6 and *σ* = 0.1. (**a**) Synchronous exclusion; (**b**) asynchronous exclusion. In the synchronous exclusion situation, the system reaches the state of L, whereas in the asynchronous exclusion situation, the system reaches the stable state of C + D + L, that is, the three strategies coexist.

**Figure 8:**
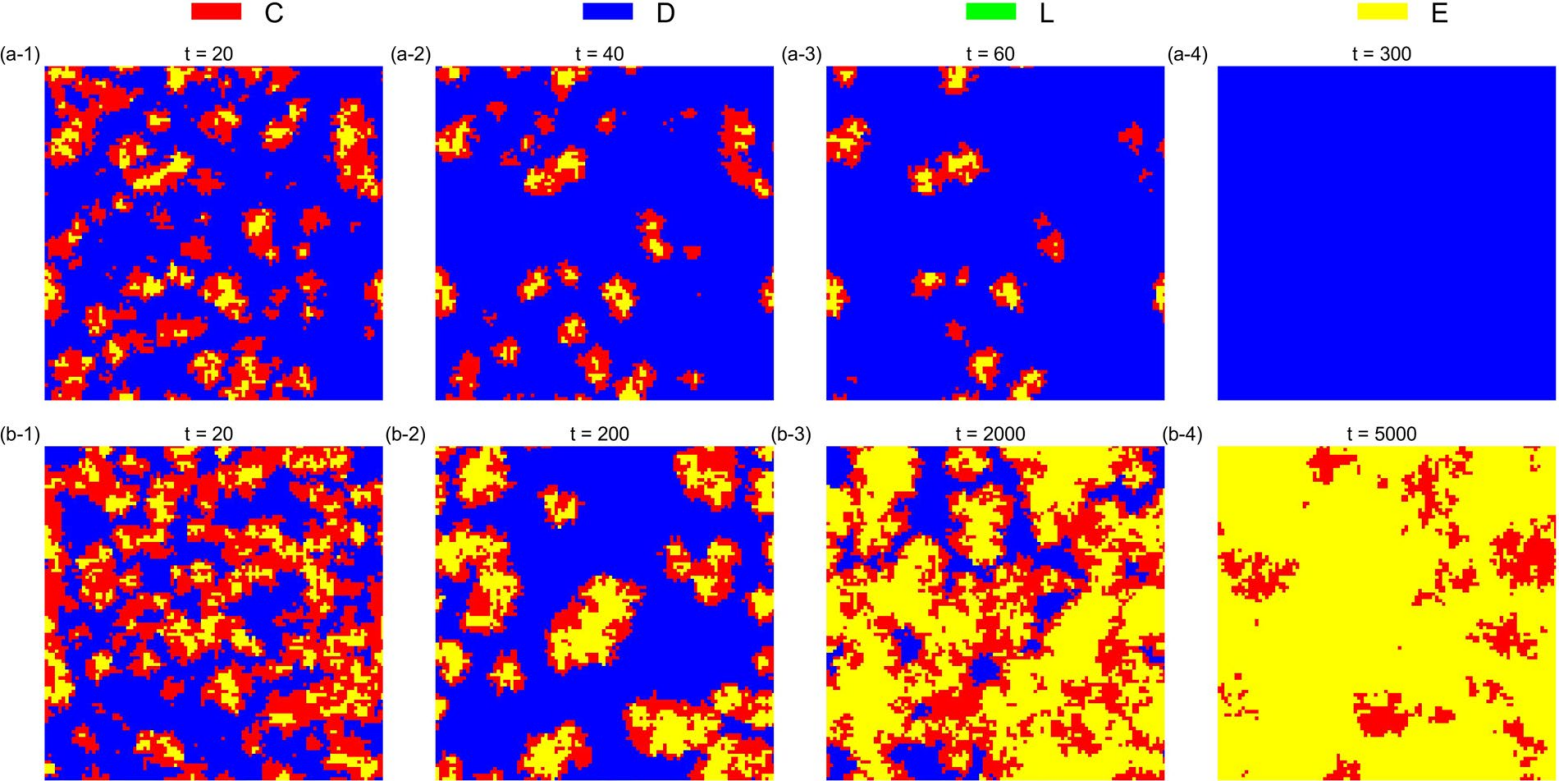
Spatiotemporal distribution of the four strategies in the PGG at *t* = 20, 40, 60, 300 MCS and *t* = 20, 200, 2000, 5000 MCS, respectively, in the two exclusion situations for one simulation, when *β* = 0.8, *r* = 2.9 and *σ* = 0.1. (**a**) Synchronous exclusion; (**b**) asynchronous exclusion. In the synchronous exclusion situation, the system reaches the state of D, whereas in the asynchronous exclusion situation, the system reaches the stable state of C + E, that is, the two cooperative strategies coexist.

**Figure 9:**
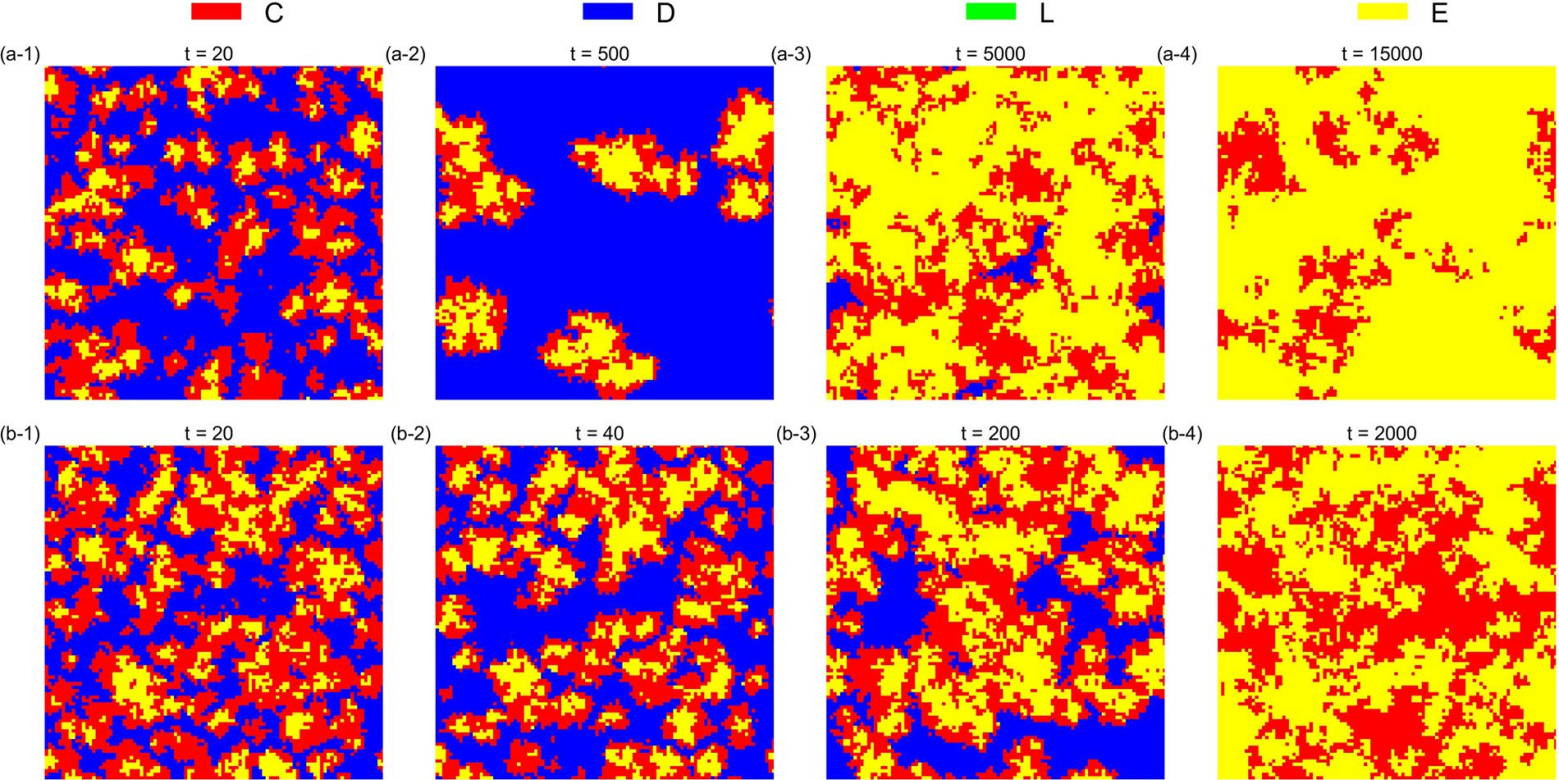
Spatiotemporal distribution of the four strategies in the PGG at *t* = 20, 500, 5000, 15000 MCS and *t* = 20, 40, 200, 2000 MCS, respectively, in the two exclusion situations for one simulation, when *β* = 0.8, *r* = 3.1 and *σ* = 0.1. (**a**) Synchronous exclusion; (**b**) asynchronous exclusion. In both exclusion situations, the system reaches the stable state of C + E, that is, the two cooperative strategies coexist.

The simulation results also verify that the asynchronous exclusion works better than synchronous exclusion for promoting cooperation in the structured population. When *β* is small (*β* = 0.1), the advantage of the asynchronous exclusion is not evident. In this situation, a common *r* interval (3.1, 3.7) emerges in the two situations, so that the *D* strategy can emerge and the frequency of *D* peaks when *r* = 3.5 after the system becomes stable. Conversely, when *β* is large (*β* = 0.8), the asynchronous exclusion mechanism has a significant advantage mainly in the middle interval of *r*, the range of which is different from that in the well-mixed population. Two different *r* intervals (2.2, 2.9) and (2.5, 3.1) exist, which correspond to the asynchronous and synchronous exclusions, respectively, so that the *D* strategy can emerge. To observe the evolution of the spatial distribution of strategies under the two exclusion mechanisms, we choose four parameter combinations for comparison. Figures 6–9 show the spatiotemporal distribution of the four strategies in the PGG at different Monte Carlo steps (MCS) in the two exclusion situations.

## Discussion

We verified that the asynchronous exclusion mechanism is indeed better than the synchronous exclusion mechanism for promoting cooperation in the well-mixed and structured populations. The benefits of the asynchronous exclusion are measured by comparing the probability that the system chooses the cooperative states in the two situations. In the well-mixed population cases, only when the investment amplification factor is small and the probability of exclusion success is high will the asynchronous exclusion mechanism have a relatively large advantage in promoting cooperation. However, in the structured population cases, the range of the investment amplification factor, in which the asynchronous exclusion mechanism has relatively large advantages in promoting cooperation, is somewhat different and is mainly in the middle of the interval under our parameters.

The three mechanisms of population structure, voluntary participation and exclusion can promote the evolution of cooperation. However, when these mechanisms exist simultaneously, we corroborate that within our parameters, an interval of *r* emerges, in which a large proportion of individuals choose defection after the system becomes stable. Figure 5-(b) illustrates that when *β* = 0.8, the defection frequency reaches a peak value of approximately 0.4 at around *r* = 2.7 in the asynchronous exclusion situation; and reaches a peak value of roughly 0.8 at around *r* = 3.0 in the synchronous exclusion situation. The figure further depicts that in the well-mixed population under the same parameters, the frequency of defection is nearly zero. Thus, when non-participation and exclusion strategies exist, the population structure does not necessarily promote cooperation compared with the well-mixed population for some parameter combinations. These results can further enrich the existing conclusions on the voluntary and exclusion mechanisms for the evolution of cooperation. When the population has a heterogeneous network structure, the relevant conclusions need further verification, and we will explore this issue in the subsequent research.

## Methods

### Evolutionary dynamics in a finite size and well-mixed population

Suppose a finite size population consisting *M* individuals. Let variables *X*, *Y*, *Z* and *W* denote the numbers of cooperators, defectors, loners and excluders in the population, respectively. Each time step, *N* individuals are sampled randomly from the population to play the PGG. Let variables *i*, *j*, *k* and *l* denote the number of cooperators, defectors, loners and excluders, respectively, in a sampled group.

In the synchronous exclusion situation, the expected payoffs of the cooperation, defection, loner and exclusion type strategies are as follows. The details of analysis can refer to Supplementary Information.

$$\begin{array}{rcl}{\pi }_{C}^{(X,Y,Z,W)} & = & \sum _{l=1}^{N-1}\sum _{k=0}^{N-1-l}\sum _{j=0}^{N-1-l-k}\frac{(\begin{array}{c}W\\ l\end{array})(\begin{array}{c}Z\\ k\end{array})(\begin{array}{c}Y\\ j\end{array})(\begin{array}{c}M-1-W-Z-Y\\ N-1-l-k-j\end{array})}{(\begin{array}{c}M-1\\ N-1\end{array})}\\  &  & \times \,\sum _{s=0}^{j}(\begin{array}{c}j\\ s\end{array}){{p}_{1}}^{s}{(1-{p}_{1})}^{j-s}[\frac{r(N-k-j)}{N-k-j+s}-1]\\  &  & +\,\frac{(\begin{array}{c}M-1-W\\ N-1\end{array})}{(\begin{array}{c}M-1\\ N-1\end{array})}\sum _{k=0}^{N-2}\sum _{j=0}^{N-1-k}\frac{(\begin{array}{c}Z\\ k\end{array})(\begin{array}{c}Y\\ j\end{array})(\begin{array}{c}M-1-W-Z-Y\\ N-1-k-j\end{array})}{(\begin{array}{c}M-1-W\\ N-1\end{array})}\\  &  & \times \,[\frac{r(N-k-j)}{N-k}-1]+\frac{(\begin{array}{c}Z\\ N-1\end{array})}{(\begin{array}{c}M-1\\ N-1\end{array})}\sigma (X\ne 0),\end{array}$$

where *p*_1_ = (1 − *β*)^*l*^.

$$\begin{array}{rcl}{\pi }_{D}^{(X,Y,Z,W)} & = & \sum _{l=1}^{N-1}\sum _{k=0}^{N-1-l}\sum _{j=0}^{N-1-l-k}\frac{(\begin{array}{c}W\\ l\end{array})(\begin{array}{c}Z\\ k\end{array})(\begin{array}{c}Y-1\\ j\end{array})(\begin{array}{c}M-W-Z-Y\\ N-1-l-k-j\end{array})}{(\begin{array}{c}M-1\\ N-1\end{array})}\\  &  & \times \,{p}_{1}\sum _{s=0}^{j}(\begin{array}{c}j\\ s\end{array}){{p}_{1}}^{s}{(1-{p}_{1})}^{j-s}[\frac{r(N-k-j-1)}{N-k-j+s}]\\  &  & +\,\frac{(\begin{array}{c}M-1-W\\ N-1\end{array})}{(\begin{array}{c}M-1\\ N-1\end{array})}\sum _{k=0}^{N-2}\sum _{j=0}^{N-1-k}\frac{(\begin{array}{c}Z\\ k\end{array})(\begin{array}{c}Y-1\\ j\end{array})(\begin{array}{c}M-W-Z-Y\\ N-1-k-j\end{array})}{(\begin{array}{c}M-1-W\\ N-1\end{array})}\\  &  & \times \,[\frac{r(N-k-j-1)}{N-k}]+\frac{(\begin{array}{c}Z\\ N-1\end{array})}{(\begin{array}{c}M-1\\ N-1\end{array})}\sigma \,(Y\ne 0),\end{array}$$

$${\pi }_{L}^{(X,Y,Z,W)}=\sigma \,(Z\ne 0),$$

$$\begin{array}{rcl}{\pi }_{E}^{(X,Y,Z,W)} & = & \sum _{l=0}^{N-1}\sum _{k=0}^{N-1-l}\sum _{j=0}^{N-1-l-k}\frac{(\begin{array}{c}W-1\\ l\end{array})(\begin{array}{c}Z\\ k\end{array})(\begin{array}{c}Y\\ j\end{array})(\begin{array}{c}M-W-Z-Y\\ N-1-l-k-j\end{array})}{(\begin{array}{c}M-1\\ N-1\end{array})}\sum _{s=0}^{j}(\begin{array}{c}j\\ s\end{array})\\  &  & \times \,{{p}_{2}}^{s}{(1-{p}_{2})}^{j-s}[\frac{r(N-k-j)}{N-k-j+s}-1-{c}_{E}j]\\  &  & -\frac{(\begin{array}{c}Z\\ N-1\end{array})}{(\begin{array}{c}M-1\\ N-1\end{array})}(r-1)+\frac{(\begin{array}{c}Z\\ N-1\end{array})}{(\begin{array}{c}M-1\\ N-1\end{array})}\sigma \,(W\ne 0),\end{array}$$

where *p*_2_ = (1 − *β*)^*l*+1^.

In the asynchronous exclusion situation, the expected payoffs of cooperators, defectors and loners remain the same, but the expected payoff of exclusion-type strategy becomes

$$\begin{array}{rcl}{\pi }_{E}^{(X,Y,Z,W)} & = & \sum _{l=0}^{N-1}\sum _{k=0}^{N-1-l}\sum _{j=0}^{N-1-l-k}\frac{(\begin{array}{c}W-1\\ l\end{array})(\begin{array}{c}Z\\ k\end{array})(\begin{array}{c}Y\\ j\end{array})(\begin{array}{c}M-W-Z-Y\\ N-1-l-k-j\end{array})}{(\begin{array}{c}M-1\\ N-1\end{array})}\sum _{s=0}^{j}(\begin{array}{c}j\\ s\end{array}){{p}_{2}}^{s}\\  &  & \times \,{(1-{p}_{2})}^{j-s}[\frac{r(N-k-j)}{N-k-j+s}-1-{c}_{R}j]\\  &  & -\frac{(\begin{array}{c}Z\\ N-1\end{array})}{(\begin{array}{c}M-1\\ N-1\end{array})}(r-1)+\frac{(\begin{array}{c}Z\\ N-1\end{array})}{(\begin{array}{c}M-1\\ N-1\end{array})}\sigma \,(W\ne 0).\end{array};$$

*c*_*R*_ is the expected unit expulsion cost in the asynchronous exclusion situation, where ${c}_{{\rm{R}}}=\frac{1-{(1-\beta )}^{l+1}}{(l+1)\beta }{c}_{E}$.

When *X*, *Y*, *Z* and *W* take zero, respectively, the corresponding ${\pi }_{C}^{(0,Y,Z,W)}$, ${\pi }_{D}^{(X,0,Z,W)}$, ${\pi }_{L}^{(X,Y,0,W)}$ and ${\pi }_{E}^{(X,Y,Z,0)}$ make no sense. In this situation, the payoff of each type strategy is defined as the average payoff of the population.

In the evolution, different types of strategies will mutually transfer based on their relative payoffs. We use the concept of transfer rate ${p}_{{s}_{1}\to {s}_{2}}^{(X,Y,Z)}=\varepsilon +\kappa \cdot {({\pi }_{{s}_{2}}^{(X,Y,Z)}-{\pi }_{{s}_{1}}^{(X,Y,Z)})}^{+}$, where ${f}^{+}=\,{\rm{\max }}(f,0)$, *ε* > 0 is a small positive number, *κ* > 0 is a parameter to describe individuals’ reaction speed to the environment in their decision, *s*_1_, *s*_2_ ∈ {*C*, *D*, *L*, *E*}, *s*_1_ ≠ *s*_2_ to describe the relative rate of transfer between different strategies, which is different from the transition probability (such as given by the Fermi function) of individuals in a structured population. The transfer rate is a macro-indicator that describes the mutual transfer intensity between four different strategies in the well-mixed system, whereas the agent-based transition probability is a micro-indicator to describe how individuals change their strategies. In fact, we can use the transfer rate to understand how individuals evolve (change their strategies). Each time interval *t* (*t* is adequately small), one of the four kinds of strategies is chosen. Without loss of generality, we assume that it is a *C* strategy. Then the probabilities of its transfer to the *D*, *L* and *E* strategies are ${p}_{C\to D}^{(X,Y,Z)}t+o(t)$, ${p}_{C\to L}^{(X,Y,Z)}t+o(t)$ and ${p}_{C\to E}^{(X,Y,Z)}t+o(t)$, respectively, and with probability $1-\sum _{S\in \{D,L,E\}}{p}_{C\to S}^{(X,Y,Z)}t-o(t)$ to remain the same, where (*x*, *y*, *z*) is the state of the system and *o*(*t*) is a high order infinitesimal of *t* when *t* is adequately small. Changes in individual strategies will result in changes in the state of the system. The evolution of the system can be described as an ergodic multi-dimensional Markov process. Based on the limit distribution of the process, we can obtain the stochastic stable states of the system and their corresponding limit probabilities. The details of the Markov-process-based dynamics and the definition of stochastic stable equilibrium can refer to Supplementary Information.

### Evolutionary dynamics in a structured population

We consider a 100 × 100 square lattice with periodic boundary conditions. Initially, all the four strategy types (*C*, *D*, *L* and *E*) are distributed uniformly at random on the network. Let ${\pi }_{{s}_{i}}$ denote the cumulative payoff of individual *i* (with strategy *s*_*i*_) participating in five rounds of the PGG. The system evolves according to the following rules. Each time, an individual *i* is randomly selected. Then, individual *i* chooses one of its neighbors at random. The chosen individual *j* imitates the strategy of individual *i* with probability $p({s}_{i}\to {s}_{j})=\frac{1}{1+\exp [({\pi }_{{s}_{j}}-{\pi }_{{s}_{i}})/\tau ]}$, where *τ* is a parameter indicating the intensity of noise. When *τ* → 0, individual *j* imitates the strategy of individual *i* if and only if ${\pi }_{{s}_{i}} > {\pi }_{{s}_{j}}$. When *τ* → + ∞, whether individual *j* imitates the strategy of individual *i* is completely random. In our simulation, we fix parameter *τ* to 0.1 and repeat the above process 10000 times as a round of iterations to ensure that each node has an opportunity to adjust its strategy on average in one iteration. The system can be stabilized after a sufficiently large number of iterations.

## Supplementary information

Supplementary Information

## References

[1] Tucker, A. W. A two-person dilemma. *Readings in games and information*, 7–8 (1950).

[2] Hardin G (1968). The tragedy of the commons. Science.

[3] Hamburger H (1973). N-person Prisoner’s Dilemma. Journal of Mathematical Sociology.

[4] Johnson DDP, Stopka P, Knights S (2003). The puzzle of human cooperation. Nature.

[5] Hasson R, Lofgren A, Visser M (2010). Climate change in a public goods game Investment decision in mitigation versus adaptation. Ecolog. Econ..

[6] Milinski, M., Hilbe, C., Semmann, D., Sommerfeld, R. & Marotzke, J. Humans choose representatives who enforce cooperation in social dilemmas through extortion. *Nat Commun* **7** (2016). 10.1038/ncomms10915 PMC4786683 26948250

[7] Peysakhovich A, Rand DG (2016). Habits of Virtue: Creating Norms of Cooperation and Defection in the Laboratory. Manage Sci.

[8] Ernst F, Urs F (2003). The nature of human altruism. Nature.

[9] Yang L, Xu Z, Zhang L, Yang D (2018). Strategy intervention in spatial voluntary public goods games. Epl.

[10] Perc M (2017). Statistical physics of human cooperation. Phys. Rep..

[11] Kennedy D, Norman C (2005). What don’t we know?. Science.

[12] Pennisi E (2005). How did cooperative behavior evolve. Science.

[13] Burton-Chellew MN, West SA (2012). Pseudocompetition among groups increases human cooperation in a public-goods game. Anim Behav.

[14] Rosas A (2010). Evolutionary game theory meets social science: Is there a unifying rule for human cooperation?. J. Theor. Biol..

[15] Perc M (2016). Phase transitions in models of human cooperation. Phys. Lett. A.

[16] West SA, Kummerli R, Burton-Chellew MN, Ross-Gillespie A (2010). Resistance to extreme strategies, rather than prosocial preferences, can explain human cooperation in public goods games. Proc. Natl. Acad. Sci. USA.

[17] O’Gorman R, Henrich J, Van Vugt M (2009). Constraining free riding in public goods games: designated solitary punishers can sustain human cooperation. Pro. R. Soc. B.

[18] Wedekind C, Milinski M (2000). Cooperation through image scoring in humans. Science.

[19] Fudenberg D, Rand DG, Dreber A (2012). Slow to Anger and Fast to Forgive: Cooperation in an Uncertain World. Amer. Econ. Rev..

[20] Hauser OP, Rand DG, Peysakhovich A, Nowak MA (2014). Cooperating with the future. Nature.

[21] Allen B (2017). Evolutionary dynamics on any population structure. Nature.

[22] Lieberman E, Hauert C, Nowak MA (2005). Evolutionary dynamics on graphs. Nature.

[23] Nowak MA, Robert MM (1992). Evolutionary games and spatial chaos. Nature.

[24] Nowak MA, Sasaki A, Taylor C, Fudenberg D (2004). Emergence of cooperation and evolutionary stability in finite populations. Nature.

[25] Ohtsuki H, Iwasa Y, Nowak MA (2009). Indirect reciprocity provides a narrow margin of efficiency for costly punishment. Nature.

[26] Nowak MA, Sigmund K (2005). Evolution of indirect reciprocity. Nature.

[27] Ohtsuki H, Hauert C, Lieberman E, Nowak MA (2006). A simple rule for the evolution of cooperation on graphs and social networks. Nature.

[28] Traulsen A, Nowak MA (2006). Evolution of cooperation by multilevel selection. Proc. Natl. Acad. Sci. USA.

[29] Hauert C, Traulsen A, Brandt H, Nowak MA, Sigmund K (2007). Via freedom to coercion: The emergence of costly punishment. Science.

[30] Nowak MA (2006). Five rules for the evolution of cooperation. Science.

[31] Rand DG, Dreber A, Ellingsen T, Fudenberg D, Nowak MA (2009). Positive Interactions Promote Public Cooperation. Science.

[32] Quan, J., Zhou, Y., Zhang, M., Tang, C. & Wang, X. The Impact of Heterogeneous Scale Return Coefficient between Groups on the Emergence of Cooperation in Spatial Public Goods Game. *J. Stat. Mech*., 043402 (2019).

[33] Sefton M, Shupp R, Walker JM (2007). The effect of rewards and sanctions in provision of public goods. Econ Inq.

[34] Sigmund K, Hauert C, Nowak MA (2001). Reward and punishment. Proc. Natl. Acad. Sci. USA.

[35] Quan J, Chu Y, Liu W, Wang X, Yang X (2018). Stochastic evolutionary public goods game with first and second order costly punishments in finite populations. Chin Phys B.

[36] Quan J, Yang X, Wang X (2018). Continuous spatial public goods game with self and peer punishment based on Particle Swarm Optimization. Phys. Lett. A.

[37] Quan J, Liu W, Chu Y, Wang X (2017). Stochastic evolutionary voluntary public goods game with punishment in a Quasi-birth-and-death process. Sci. Rep..

[38] Fehr E, Gachter S (2000). Cooperation and punishment in public goods experiments. Amer. Econ. Rev..

[39] Zhou Y, Jiao P, Zhang Q (2017). Second-party and third-party punishment in a public goods experiment. Appl Econ Lett.

[40] Szolnoki A, Perc M (2010). Reward and cooperation in the spatial public goods game. Epl.

[41] Sasaki T, Unemi T (2011). Replicator dynamics in public goods games with reward funds. J. Theor. Biol..

[42] dos Santos, M. The evolution of anti-social rewarding and its countermeasures in public goods games. *Pro. R. Soc. B* **282** (2015). 10.1098/rspb.2014.1994 PMC4262171 25429015

[43] Sasaki T, Uchida S, Chen X (2015). Voluntary rewards mediate the evolution of pool punishment for maintaining public goods in large populations. Sci. Rep..

[44] Fowler JH (2005). Altruistic punishment and the origin of cooperation. Proc. Natl. Acad. Sci. USA.

[45] Ozono H, Jin N, Watabe M, Shimizu K (2016). Solving the second-order free rider problem in a public goods game: An experiment using a leader support system. Sci. Rep..

[46] Ye H (2016). Increasing returns to scale: The solution to the second-order social dilemma. Sci. Rep..

[47] Szolnoki A, Perc M (2013). Effectiveness of conditional punishment for the evolution of public cooperation. J. Theor. Biol..

[48] Chen X, Szolnoki A, Perc M (2014). Probabilistic sharing solves the problem of costly punishment. New J. Phys..

[49] Perc M, Szolnoki A (2012). Self-organization of punishment in structured populations. New J. Phys..

[50] Yamamoto H, Okada I (2016). How to keep punishment to maintain cooperation: Introducing social vaccine. Physica a.

[51] D’Orsogna MR, Short MB, Brantingham PJ (2010). Cooperation and punishment in an adversarial game: How defectors pave the way to a peaceful society. Phys. Rev. E.

[52] Helbing D, Szolnoki A, Perc M, Szabó G (2010). Defector-accelerated cooperativeness and punishment in public goods games with mutations. Phys. Rev. E.

[53] Szolnoki A, Szabo G, Czako L (2011). Competition of individual and institutional punishments in spatial public goods games. Phys. Rev. E.

[54] Szolnoki A, Szabo G, Perc M (2011). Phase diagrams for the spatial public goods game with pool punishment. Phys. Rev. E.

[55] Brandt H, Hauert C, Sigmund K (2003). Punishment and reputation in spatial public goods games. Proc. R. Soc. London, Ser. B.

[56] Chen X, Sasaki T, Perc M (2015). Evolution of public cooperation in a monitored society with implicated punishment and within-group enforcement. Sci. Rep..

[57] Perc M, Szolnoki A (2015). A double-edged sword: Benefits and pitfalls of heterogeneous punishment in evolutionary inspection games. Sci. Rep..

[58] Wang Z, Xia CY, Meloni S, Zhou CS, Moreno Y (2013). Impact of Social Punishment on Cooperative Behavior in Complex Networks. Sci. Rep..

[59] Helbing D, Szolnoki A, Perc M, Szabó G (2010). Punish, but not too hard: how costly punishment spreads in the spatial public goods game. New J. Phys..

[60] Helbing D, Szolnoki A, Perc M, Szabó G (2010). Evolutionary Establishment of Moral and Double Moral Standards through Spatial Interactions. PLoS Comp. Biol..

[61] Gao J, Li Z, Cong R, Wang L (2012). Tolerance-based punishment in continuous public goods game. Physica a.

[62] Dreber A, Rand DG, Fudenberg D, Nowak MA (2008). Winners don’t punish. Nature.

[63] Nikiforakis N (2010). Feedback, punishment and cooperation in public good experiments. Games Econ. Behav..

[64] Sasaki T, Uchida S (2013). The evolution of cooperation by social exclusion. Pro. R. Soc. B.

[65] Sui XK, Wu B, Wang L (2018). Rationality alters the rank between peer punishment and social exclusion. Epl.

[66] Li K, Cong R, Wu T, Wang L (2015). Social exclusion in finite populations. Phys. Rev. E.

[67] Liu L, Chen X, Szolnoki A (2017). Competitions between prosocial exclusions and punishments in finite populations. Sci. Rep..

[68] Li K, Cong R, Wang L (2016). Cooperation induced by random sequential exclusion. Epl.

[69] Quan J, Liu W, Chu Y, Wang X (2018). Stochastic dynamics and stable equilibrium of evolutionary optional public goods game in finite populations. Physica a.

