# Supplementary material for: Benefits of asynchronous exclusion for the evolution of cooperation in stochastic evolutionary optional public goods games
Source: Sci Rep. 2019 Jun 3;9:8208. doi: 10.1038/s41598-019-44725-y (PMC6547755; doi:10.1038/s41598-019-44725-y)
Supplement: Supplementary file 1 — Supplementary Information [file 41598_2019_44725_MOESM1_ESM.pdf]

Supplementary Information: Benefits of asynchronous exclusion for the evolution of cooperation in stochastic evolutionary optional public goods games

Ji Quan[1], Junjun Zheng[2], Xianjia Wang[2] & Xiukang Yang[1]

[1] *School of Management, Wuhan University of Technology, Wuhan 430070, China.*

[2] *School of Economics and Management, Wuhan University, Wuhan 430072, China.*

In the Supplementary Information, we provide how to calculate expected payoffs in a finite size and well-mixed population, evolutionary dynamics and the definition of stochastic stable equilibrium, and the Gauss-Seidel iterative algorithm to calculate the limit distribution of the multi-dimensional Markov process in details. Some more results are also presented in this text, including four-dimensional simplex to illustrate stochastic stable equilibriums with exclusion-type strategies and their limit probabilities, and the effect of β and population size M on the equilibrium results.

Expected Payoffs in a finite size and well-mixed population

Suppose a finite population of size M. Let variables X, Y, Z and W denote the numbers of cooperators, defectors, loners and excluders in the population, respectively. Each time, N individuals are sampled randomly from the population to participate in the PGG. Let variables i, j, k and l denote the number of cooperators, defectors, loners and excluders, respectively, in a sampled group.

We initially analyze the situation of synchronous exclusion. Let β denote the probability of success for an excluder expelling a defector. Owing to independence, the probability of a defector escaping from all excluders in the game is $p_1 = (1-\beta)^l$. In addition, let s ($0 \le s \le j$) denote the number of defectors who escape from all excluders in the group. Thereafter, s obeys the binomial distribution with parameters (j, p_1), that is $s \sim B(j, p_1)$.

When $l \ne 0$, the probability that s defectors escape from all excluders in the group is $\binom{j}{s} p_1^{\,s}(1-p_1)^{j-s}$. In this situation, the payoff of a cooperator is $\frac{r(N-k-j)}{N-k-j+s} - 1$; thus, the expected payoff of a cooperator is $\sum_{s=0}^{j}\binom{j}{s} p_1^{\,s}(1-p_1)^{j-s}[\frac{r(N-k-j)}{N-k-j+s} - 1]$.

The probability that no excluders are found in the group is $p(l=0) = \binom{M-1-W}{N-1} \Big/ \binom{M-1}{N-1}$. In this situation, the payoff of a cooperator is $\frac{r(N-k-j)}{N-k} - 1$.

The probability that all other $N-1$ individuals are loners is $\binom{Z}{N-1} \Big/ \binom{M-1}{N-1}$. In this situation, we assume that the collective investment behaviour cannot occur; thus, the payoff of any strategy (C, D, E) is σ regardless of its type.

Thus, when the numbers of cooperators, defectors, loners and excluders in the group are X, Y, Z and W, respectively, the expected payoff of a cooperation strategy is as follows:

$$\pi_C^{(X,Y,Z,W)} = \sum_{l=1}^{N-1}\sum_{k=0}^{N-1-l}\sum_{j=0}^{N-1-l-k}\frac{\binom{W}{l}\binom{Z}{k}\binom{Y}{j}\binom{M-1-W-Z-Y}{N-1-l-k-j}}{\binom{M-1}{N-1}}\sum_{s=0}^{j}\binom{j}{s}p_1^{\,s}(1-p_1)^{j-s}[\frac{r(N-k-j)}{N-k-j+s}-1]$$

$$+\frac{\binom{M-1-W}{N-1}}{\binom{M-1}{N-1}}\sum_{k=0}^{N-2}\sum_{j=0}^{N-1-k}\frac{\binom{Z}{k}\binom{Y}{j}\binom{M-1-W-Z-Y}{N-1-k-j}}{\binom{M-1-W}{N-1}}[\frac{r(N-k-j)}{N-k}-1]+\frac{\binom{Z}{N-1}}{\binom{M-1}{N-1}}\sigma\ (X\neq 0).$$

Similarly, we can obtain the expected payoffs of other strategies as follows.

$$\pi_D^{(X,Y,Z,W)} = \sum_{l=1}^{N-1}\sum_{k=0}^{N-1-l}\sum_{j=0}^{N-1-l-k}\frac{\binom{W}{l}\binom{Z}{k}\binom{Y-1}{j}\binom{M-W-Z-Y}{N-1-l-k-j}}{\binom{M-1}{N-1}}p_1\sum_{s=0}^{j}\binom{j}{s}p_1^{\,s}(1-p_1)^{j-s}[\frac{r(N-k-j-1)}{N-k-j+s}]$$

$$+\frac{\binom{M-1-W}{N-1}}{\binom{M-1}{N-1}}\sum_{k=0}^{N-2}\sum_{j=0}^{N-1-k}\frac{\binom{Z}{k}\binom{Y-1}{j}\binom{M-W-Z-Y}{N-1-k-j}}{\binom{M-1-W}{N-1}}[\frac{r(N-k-j-1)}{N-k}]+\frac{\binom{Z}{N-1}}{\binom{M-1}{N-1}}\sigma\ (Y\neq 0),$$

$$\pi_L^{(X,Y,Z,W)} = \sigma\ (Z\neq 0),$$

$$\pi_E^{(X,Y,Z,W)} = \sum_{l=0}^{N-1}\sum_{k=0}^{N-1-l}\sum_{j=0}^{N-1-l-k}\frac{\binom{W-1}{l}\binom{Z}{k}\binom{Y}{j}\binom{M-W-Z-Y}{N-1-l-k-j}}{\binom{M-1}{N-1}}\sum_{s=0}^{j}\binom{j}{s}p_2^{\,s}(1-p_2)^{j-s}[\frac{r(N-k-j)}{N-k-j+s}-1-c_E j]$$

$$-\frac{\binom{Z}{N-1}}{\binom{M-1}{N-1}}(r-1)+\frac{\binom{Z}{N-1}}{\binom{M-1}{N-1}}\sigma\ (W\neq 0),\ \text{where}\ p_2=(1-\beta)^{l+1}.$$

In the asynchronous exclusion situation, the expected payoffs of cooperators, defectors and loners remain the same, but the expected payoff of exclusion strategy becomes

$$\pi_E^{(X,Y,Z,W)} = \sum_{l=0}^{N-1}\sum_{k=0}^{N-1-l}\sum_{j=0}^{N-1-l-k}\frac{\binom{W-1}{l}\binom{Z}{k}\binom{Y}{j}\binom{M-W-Z-Y}{N-1-l-k-j}}{\binom{M-1}{N-1}}\sum_{s=0}^{j}\binom{j}{s}p_2^{\,s}(1-p_2)^{j-s}[\frac{r(N-k-j)}{N-k-j+s}-1-c_R j],$$

$$-\frac{\binom{Z}{N-1}}{\binom{M-1}{N-1}}(r-1)+\frac{\binom{Z}{N-1}}{\binom{M-1}{N-1}}\sigma\ (W\neq 0).$$

c_R is the expected unit expulsion cost in the asynchronous exclusion situation, where $c_{\mathrm{R}} = \frac{1-(1-\beta)^{l+1}}{(l+1)\beta}c_E$.

When X, Y, Z and W take zero, respectively, the corresponding $\pi_C^{(0,Y,Z,W)}$, $\pi_D^{(X,0,Z,W)}$, $\pi_L^{(X,Y,0,W)}$ and $\pi_E^{(X,Y,Z,0)}$ make no sense. In this situation, the payoff of each type strategy is defined as the average payoff of the population.

Evolutionary dynamics and stochastic stable equilibrium

A stochastic process $z(t)$ can be introduced to describe the evolutionary process of the strategies. Let $z(t)=(X(t),Y(t),Z(t),M-X(t)-Y(t)-Z(t))$ denote the number of cooperators, defectors, loners and excluders in the population at time t, respectively, and define $z(t)$ as the system state. For convenience, we abbreviate it as $(X(t),Y(t),Z(t))$. The state space of the system is $S=\{(X,Y,Z)\,|\,0\le X+Y+Z\le M; X,Y,Z\in\mathbb{N}\}$, and the number of elements in the state space is $|S|=\frac{(M+1)(M+2)(M+3)}{6}$. At each time, the individual in the population adjusts its strategy according to its expected payoff, and the adjustment of the individual's strategy leads to the change of the system state. The three assumptions, namely, inertia, myopic and mutation, in the literature[1] on the bounded rationality of individuals in the population are utilised in our model. Owing to inertia, having more than two individuals to adjust their strategies simultaneously at one time is assumed to be impossible. Myopic refers to the individual when choosing its strategy; it will only consider the current payoff, regardless of the payoff in the future. Mutation refers to the possibility that individuals may choose a non-optimal strategy with a small probability because of the complex decision-making environment and the limited nature of individual cognitive ability.

According to the above assumptions, when the system state is $(X,Y,Z)\in S$, the transfer rate of the strategy s_1 towards strategy s_2 can be described as follows:

$$p_{s_1\to s_2}^{(X,Y,Z)}=\varepsilon+\kappa\cdot(\pi_{s_2}^{(X,Y,Z)}-\pi_{s_1}^{(X,Y,Z)})^+,\ s_1,s_2\in\{C,D,L,E\}, s_1\ne s_2, \quad \text{(S1)}$$

where $f^+=\max(f,0)$, $\varepsilon>0$ is a small positive number, $\kappa>0$. For instance, when $\pi_{s_1}^{(X,Y,Z)}>\pi_{s_2}^{(X,Y,Z)}$, individuals in the (X,Y,Z) state have a more incentive to move from strategy s_2 to strategy s_1. However, owing to mutation, the transfer rate of strategy s_1 to strategy s_2 is $p_{s_1\to s_2}^{(X,Y,Z)}=\varepsilon$. Thus, parameter ε can be considered as the noise intensity in the environment, and κ can be understood as the speed at which the individual responds to the environment.

According to the system evolutionary rules and the transition rate between different strategies, after an adequately small time t, the probabilities of the system transfer from state (X,Y,Z) to states $(X-1,Y,Z+1)$, $(X-1,Y,Z)$ and $(X-1,Y+1,Z)$ are $p_{C\to L}^{(X,Y,Z)}t+o(t)$, $p_{C\to E}^{(X,Y,Z)}t+o(t)$ and $p_{C\to D}^{(X,Y,Z)}t+o(t)$, respectively; to states $(X,Y-1,Z+1)$, $(X,Y-1,Z)$ and $(X+1,Y-1,Z)$ are $p_{D\to L}^{(X,Y,Z)}t+o(t)$, $p_{D\to E}^{(X,Y,Z)}t+o(t)$ and $p_{D\to C}^{(X,Y,Z)}t+o(t)$, respectively; to states $(X,Y+1,Z-1)$, $(X,Y,Z-1)$ and $(X+1,Y,Z-1)$ are $p_{L\to D}^{(X,Y,Z)}t+o(t)$, $p_{L\to E}^{(X,Y,Z)}t+o(t)$ and $p_{L\to C}^{(X,Y,Z)}t+o(t)$, respectively; to states $(X+1,Y,Z)$, $(X,Y,Z+1)$ and $(X,Y+1,Z)$ are $p_{E\to C}^{(X,Y,Z)}t+o(t)$, $p_{E\to L}^{(X,Y,Z)}t+o(t)$ and $p_{E\to D}^{(X,Y,Z)}t+o(t)$, respectively; and the probability to keep the same state is $1-\sum_{\substack{s_1,s_2\in\{C,D,L,E\}\\ s_1\ne s_2}} p_{s_1\to s_2}^{(X,Y,Z)}t-o(t)$, where $o(t)$ is a high-order infinitesimal of t when t is adequately small.

Let $I=(X,Y,Z)$, $I'=(X',Y',Z')$. Owing to time homogeneity, let $p_{I,I'}(t)$ denote the probability of the system that transfers from state I to state I' after time t. That is,

$$p_{I,I'}(t)=p\{z(s+t)=(i',j',k')\,|\,z(s)=(i,j,k)\}, \forall s>0. \quad \text{(S2)}$$

Whilst $\varepsilon>0$, this process is ergodic, according to the properties of the stochastic process, when $t\to+\infty$, the limit of $p_{I,I'}(t)$ exists, and it is independent from the initial state I. Let

$$\lim_{t\to+\infty} p_{I,I'}(t) = v_{I'}^{\varepsilon}. \quad \text{(S3)}$$

Thereafter, $v_{I'}^{\varepsilon}$, which is the limit distribution of the stochastic process, reaches an arbitrary state I' $(I' \in S)$ when the system noise is ε. According to the limit distribution, determining the evolutionary stable state of the system under arbitrary noise intensity is possible. Furthermore, when the noise parameters are slowly reduced to zero, let

$$\lim_{\varepsilon\to 0^+} v_{I'}^{\varepsilon} = v_{I'}. \quad \text{(S4)}$$

According to $v_{I'}$, we can determine the limit state of the system and its probability distribution when the system noise is vanishing. According to Young's description in reference[2], state $I' \in S$ is stochastically stable if and only if $v_{I'} > 0$.

The Gauss-Seidel iterative algorithm

The Gauss-Seidel iterative algorithm proposed in the literature[3] can be utilised to calculate the limit probabilities of the multi-dimensional Markov process. The algorithm can be summarized as follows.

Input：infinitesimal generator matrix $\mathbf{Q}=(q_{ij})$

Output: the limit distribution of each state $\mathbf{x}=(x_i)$

Choose an initial guess $\mathbf{x}=\mathbf{e}_1=(1,0,\ldots,0)$ to the solution
repeat until convergence
  for i from 1 to n do
    $\sigma \leftarrow 0$
    for j from 1 to n do
      if $j \neq i$ then
        $\sigma \leftarrow \sigma + q_{ij}x_j$
      end if
    end (j-loop)
    $x_i \leftarrow -\dfrac{1}{q_{ii}}\sigma$
  end (i-loop)
    check if convergence is reached
end (repeat)

The infinitesimal generator matrix $\mathbf{Q}=(q_{ij})$ is as follows (the blank of each matrix equals zero).

$$\mathbf{Q}=\begin{pmatrix} A_0 & C_0 & & & \\ B_1 & A_1 & C_1 & & \\ & \ddots & \ddots & \ddots & \\ & & B_{M-1} & A_{M-1} & C_{M-1} \\ & & & B_M & A_M \end{pmatrix}, \text{ where}$$

$$A_i=\begin{pmatrix} D_{i,0} & F_{i,0} & & & \\ E_{i,1} & D_{i,1} & F_{i,1} & & \\ & \ddots & \ddots & \ddots & \\ & & E_{i,M-i-1} & D_{i,M-i-1} & F_{i,M-i-1} \\ & & & E_{i,M-i} & D_{i,M-i} \end{pmatrix}, \text{ where}$$

$$
D_{i,j}=\begin{pmatrix}
d_{i,\mathrm{j},0} & p_{E\to L}^{(\mathrm{i,j},0)} & & & \\
p_{L\to E}^{(\mathrm{i,j},1)} & d_{i,\mathrm{j},1} & p_{E\to L}^{(\mathrm{i,j},1)} & & \\
 & \ddots & \ddots & \ddots & \\
 & & p_{L\to E}^{(\mathrm{i,j,M-i-j-1})} & d_{i,\mathrm{j,M-i-j-1}} & p_{E\to L}^{(\mathrm{i,j,M-i-j-1})} \\
 & & & p_{L\to E}^{(\mathrm{i,j,M-i-j})} & d_{i,\mathrm{j,M-i-j}}
\end{pmatrix},
$$

$$
F_{i,j}=\begin{pmatrix}
p_{E\to D}^{(\mathrm{i,j},0)} & & & \\
p_{L\to D}^{(\mathrm{i,j},1)} & p_{E\to D}^{(\mathrm{i,j},1)} & & \\
 & p_{L\to D}^{(\mathrm{i,j},2)} & \ddots & \\
 & & \ddots & p_{E\to D}^{(\mathrm{i,j,M-i-j-1})} \\
 & & & p_{L\to D}^{(\mathrm{i,j,M-i-j})}
\end{pmatrix},
E_{i,j}=\begin{pmatrix}
p_{D\to E}^{(\mathrm{i,j},0)} & p_{D\to L}^{(\mathrm{i,j},0)} & & & \\
 & p_{D\to E}^{(\mathrm{i,j},1)} & p_{D\to L}^{(\mathrm{i,j},1)} & & \\
 & & \ddots & \ddots & \\
 & & & p_{D\to E}^{(\mathrm{i,j,M-i-j})} & p_{D\to L}^{(\mathrm{i,j,M-i-j})}
\end{pmatrix};
$$

and $B_i=\begin{pmatrix}
G_{i,0} & H_{i,0} & & & \\
 & G_{i,1} & H_{i,1} & & \\
 & & \ddots & \ddots & \\
 & & & G_{i,\mathrm{M-i}} & H_{i,\mathrm{M-i}}
\end{pmatrix}$, where

$$
G_{i,j}=\begin{pmatrix}
p_{C\to E}^{(\mathrm{i,j},0)} & p_{C\to L}^{(\mathrm{i,j},0)} & & & \\
 & p_{C\to E}^{(\mathrm{i,j},1)} & p_{C\to L}^{(\mathrm{i,j},1)} & & \\
 & & \ddots & \ddots & \\
 & & & p_{C\to E}^{(\mathrm{i,j,M-i-j})} & p_{C\to L}^{(\mathrm{i,j,M-i-j})}
\end{pmatrix},
H_{i,j}=\begin{pmatrix}
p_{C\to D}^{(\mathrm{i,j},0)} & & & \\
 & p_{C\to D}^{(\mathrm{i,j},1)} & & \\
 & & \ddots & \\
 & & & p_{C\to D}^{(\mathrm{i,j,M}-i-j)}
\end{pmatrix};
$$

and $C_i=\begin{pmatrix}
K_{i,0} & & & \\
I_{i,1} & K_{i,1} & & \\
 & I_{i,2} & \ddots & \\
 & & \ddots & K_{i,\mathrm{M}-i-1} \\
 & & & I_{i,\mathrm{M-i}}
\end{pmatrix}$, where

$$
K_{i,j}=\begin{pmatrix}
p_{E\to C}^{(\mathrm{i,j},0)} & & & \\
p_{L\to C}^{(\mathrm{i,j},1)} & p_{E\to C}^{(\mathrm{i,j},1)} & & \\
 & p_{L\to C}^{(\mathrm{i,j},2)} & \ddots & \\
 & & \ddots & p_{E\to C}^{(\mathrm{i,j,M-i-j-1})} \\
 & & & p_{L\to C}^{(\mathrm{i,j,M-i-j})}
\end{pmatrix},\quad
I_{i,j}=\begin{pmatrix}
p_{D\to C}^{(\mathrm{i,j},0)} & & & \\
 & p_{D\to C}^{(\mathrm{i,j},1)} & & \\
 & & \ddots & \\
 & & & p_{D\to C}^{(\mathrm{i,j,M}-i-j)}
\end{pmatrix}.
$$

The $d_{i,j,k}$ ($i,j,k=0,1,\ldots,M$ *and* $i+j+k\le M$) of $\mathbf{Q}=(\mathrm{q}_{ij})$ can be obtained by equations $\mathbf{Q1}=\mathbf{0}$, where $\mathbf{1}$ is the column vector with all components equal to one, and $\mathbf{0}$ is the column vector with all components equal to zero.

Results in details

In the finite size and well-mixed population, only the states of $(0,M,0,0)$, $(0,0,M,0)$, $(0,1,M-1,0)$, $(0,0,M-1,1)$ and $(i,0,0,M-i)$ $(0\le i\le M)$ may be stochastically stable. States $(0,0,M,0)$, $(0,1,M-1,0)$ and $(0,0,M-1,1)$ denote 'All L'; State $(0,M,0,0)$ denotes 'All D' ; States $(i,0,0,M-i)$ $(0\le i\le M)$ denote 'C+E'. We initially use the four-dimensional simplex (S4) method to illustrate the stochastic stable states and their probabilities. Figures S1 and S2 show the results in the asynchronous exclusion situation for six different parameter combinations. In addition, Figure S1 elucidates that when parameters (r,β) are fixed at $(3.5,0.1)$, the increase in σ can effectively prevent the replication of defection in the population. The probability of the system

choosing the ‘All D’ state is reduced from 0.878 (when $\sigma = 0.1$) to 0.159 (when $\sigma = 0.5$) and then to zero (when $\sigma = 1$). However, at this time, given that the probability of successful exclusion is small ($\beta = 0.1$), the probability of the system selecting the “All L” state will slowly increase with the increase in σ, increasing from zero (when $\sigma = 0.1$) to 0.078 (when $\sigma = 0.5$) and then to 0.185 (when $\sigma = 1$). Figure S2 shows another scenario when parameters (r, σ) are fixed at $(2.5, 0.5)$, in which case the increase in β can effectively promote the replication of cooperation in the population. Furthermore, the probability of the system selecting the ‘All D’ state is zero for any values of β; the probability of selecting the ‘All L’ state decreases with the increase in σ, from 0.879 (when $\beta = 0.1$) to 0.073 (when $\beta = 0.5$) and then to 0.038 (when $\beta = 1$); the probability of selecting the ‘C+E’ states increases with the increase in σ, from 0.121 (when $\beta = 0.1$) to 0.927 (when $\beta = 0.5$) and then to 0.962 (when $\beta = 1$).

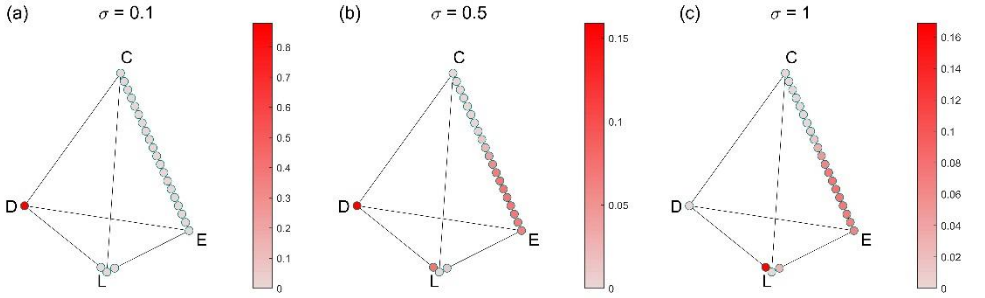

Figure S1. All stochastic stable states and their limit probabilities for fixed r=3.5 and $\beta = 0.1$ in the asynchronous exclusion. (a) σ=0.1;(b) σ=0.5;(c) σ=1. In this situation, the increase in σ can effectively prevent the replication of defection in the population. The probability of the system choosing the ‘All D’ state is reduced from 0.878 (when $\sigma = 0.1$) to 0.159 (when $\sigma = 0.5$) and then to zero (when $\sigma = 1$). However, at this time, given that the probability of successful exclusion is small ($\beta = 0.1$), the probability of the system choosing the ‘All L’ state will slowly increase with the increase in σ, increasing from zero (when $\sigma = 0.1$) to 0.078 (when $\sigma = 0.5$) and then to 0.185 (when $\sigma = 1$).

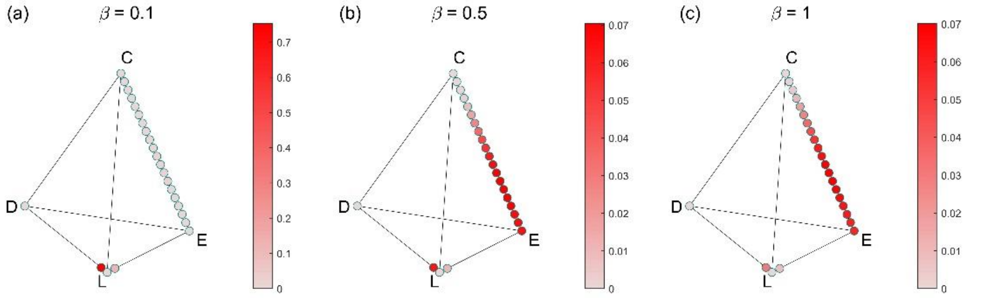

Figure S2. All stochastic stable states and their limit probabilities for fixed r=2.5 and $\sigma = 0.5$ in the asynchronous exclusion. (a) β=0.1;(b) β=0.5;(c) β=1. In this situation, the increase in β can effectively promote the replication of cooperation in the population. The probability of the system choosing the ‘All D’ state is zero for any values of β; the probability of selecting the ‘All L’ state decreases with the increase in σ from 0.879 (when $\beta = 0.1$) to 0.073 (when $\beta = 0.5$) and then to 0.038 (when $\beta = 1$); the probability of choosing the ‘C+E’ states increases with the increase in σ from 0.121 (when $\beta = 0.1$) to 0.927 (when $\beta = 0.5$) and then to 0.962 (when $\beta = 1$).

To observe the relationship between the probability of the system reaching each stable equilibrium and the values of parameters β, r, σ, we fix parameter β to some different values, and calculate the corresponding limit probability under any combinations of (r, σ). Figures S3 to S5 show the additional results.

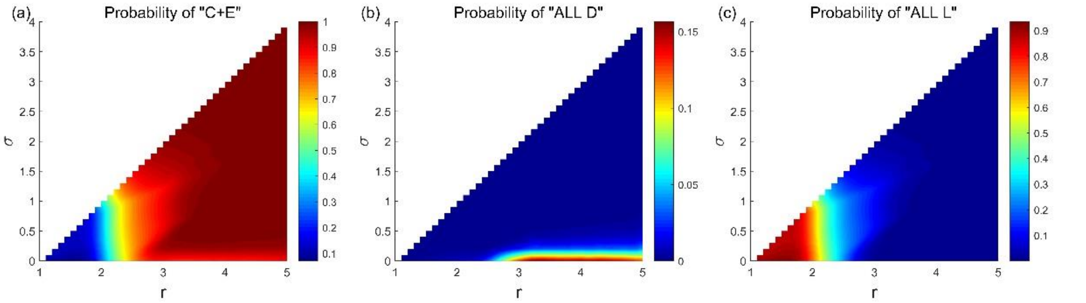

Figure S3. Limit probability of the system selecting each stochastic stable state under any parameter combinations of (r,σ) and fixed β=0.2 in the asynchronous exclusion. The increase in β has caused all three regions to shrink rapidly, and the probability of the system selecting the 'All D' state also drop rapidly when parameters (r,σ) are in the D_3 region.

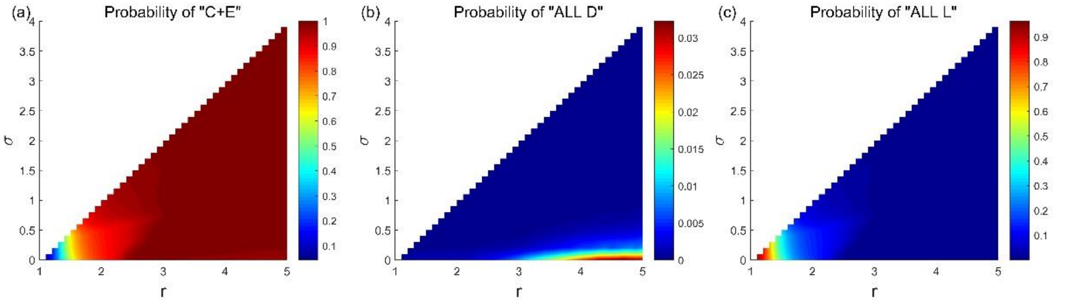

Figure S4. Limit probability of the system selecting each stochastic stable state under any parameter combinations of (r,σ) and fixed β=0.5 in the asynchronous exclusion. The increase in β further narrows the three regions.

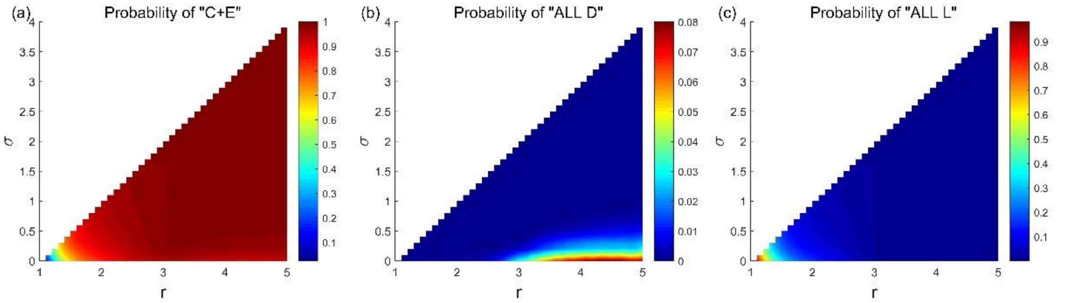

Figure S5. Limit probability of the system selecting each stochastic stable state under any parameter combinations of (r,σ) and fixed β=1 in the asynchronous exclusion. Since the cost of exclusion increases as β increases, the increase in β to 1 cannot make the three regions disappear.

To verify that our conclusions are also valid in relatively large size populations, we compare the equilibrium results across three populations of different sizes, namely, $M = 20, 50$ and 100. The corresponding comparison results are displayed in Figures S6 to S8. As shown in figure S6, when β is small (β=0.1), if r is also small, the increase in M has little effect on the probability of the equilibrium selection; if r is relatively large, the increase in M has a great impact on the equilibrium selection. In particular, there is an interval in the middle in which large M will suppress cooperation, but if r is large, large M is conducive to cooperation. When β is large (β=0.8), large M is conducive to cooperation for small r, and there is no difference when r increases to a certain value ($r \geq 2.7$ in the figure). The probability that the system selects some stable states will change significantly when M increases to 100, but for the main conclusions we present in the text, there is no essential difference between $M = 100$ and $M = 20$. Figures S7 and S8 further corroborate this judgment.

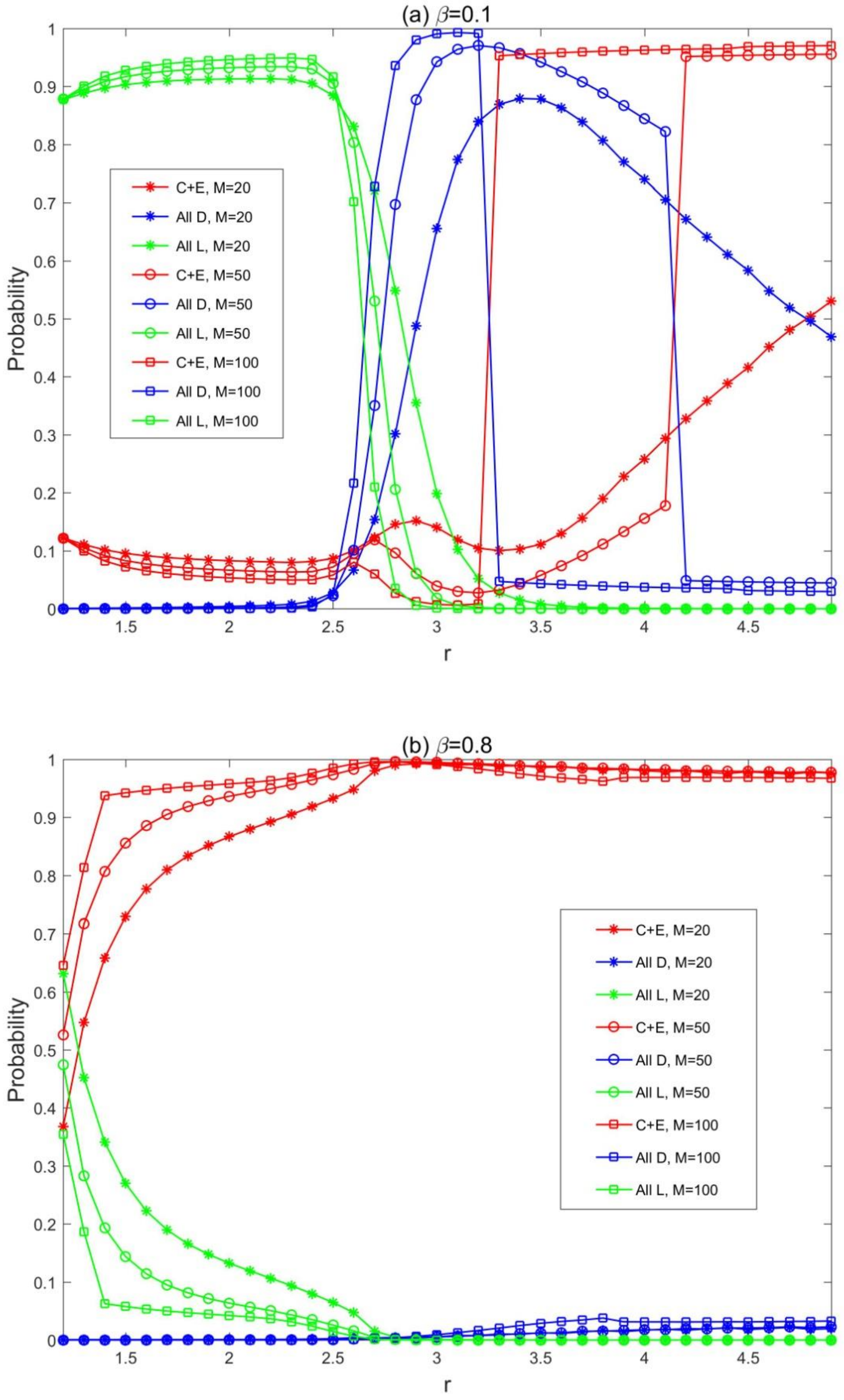

Figure S6. Relationship between the limit probability of the system selecting each type of stable states and parameter r for fixed σ=0.1 and three different population size M in the asynchronous exclusion. (a) β=0.1; (b) β=0.8. When β is small (β=0.1), if r is also small, the increase in M has little effect on the probability of the equilibrium selection; if r is relatively large, the increase in M has a great impact on the equilibrium selection. In particular, there is an interval in the middle in which large M will suppress cooperation, but if r is large,

large M is conducive to cooperation. When β is large (β=0.8), large M is conducive to cooperation for small r, and there is no difference when r increases to a certain value ($r \geq 2.7$ in the figure).

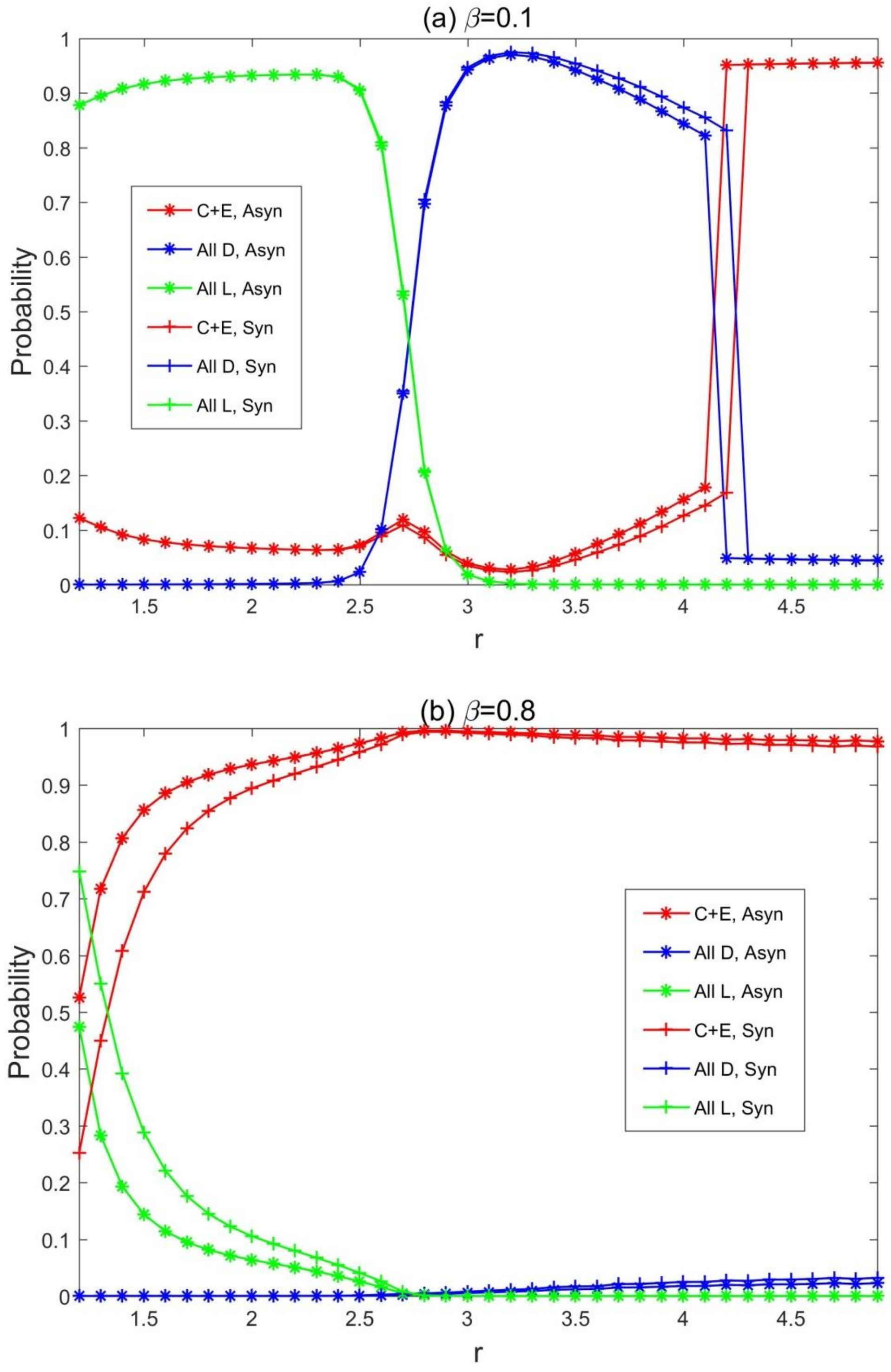

Figure S7. Relationship between the limit probability of the system selecting each type of stable states and parameter r for fixed σ=0.1 and $M = 50$ under the two exclusion mechanisms. (a) β=0.1; (b) β=0.8. There is no essential difference between the situation with $M = 20$ and it also verifies that only when r is small and β is large will the asynchronous exclusion mechanism have a relatively large advantage in promoting cooperation.

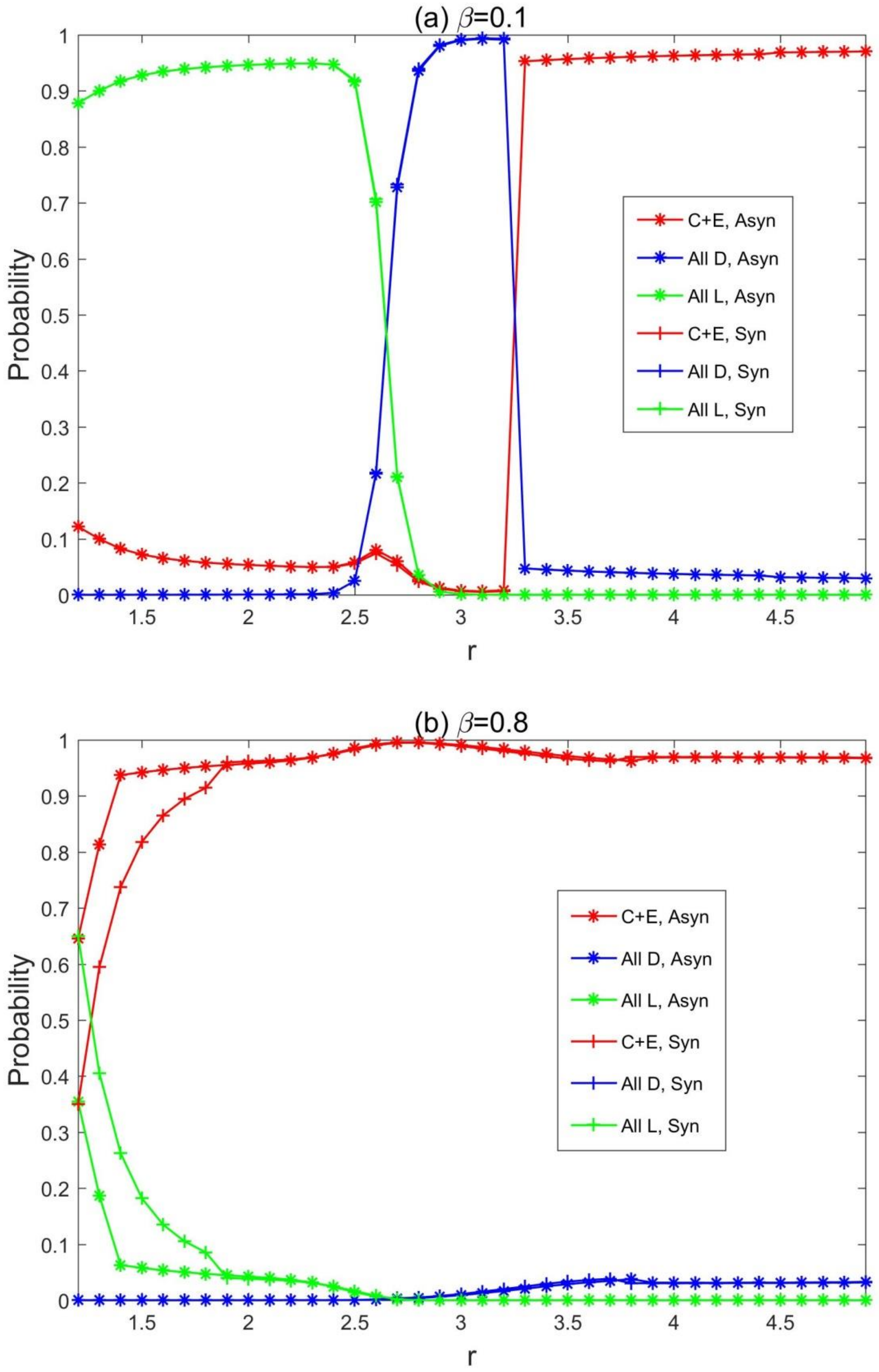

Figure S8. Relationship between the limit probability of the system selecting each type of stable states and parameter r for fixed σ=0.1 and $M = 100$ under the two exclusion mechanisms. (a) β=0.1; (b) β=0.8. There is no essential difference between the situation with $M = 20$ and it also verifies that only when r is small and β is large will the asynchronous exclusion mechanism have a relatively large advantage in promoting cooperation.

References

1. Amir, M. & Berninghaus, S. K. Another Approach to Mutation and Learning in Games. *Games Econ. Behav.* **14**, 19-43 (1996).
2. Young, P. The Evolution of Conventions. *Econometrica* **61**, 57-84 (1993).
3. Stewart, W. J. *Introduction to the Numerical Solution of Markov Chains*. (Princeton University Press, 1994).
